# Lifestyle and Occupational Factors Associated with Recurrent Stroke among Working-Age Adults in Urban Areas of Thailand

**DOI:** 10.12688/f1000research.154968.1

**Published:** 2024-11-29

**Authors:** Yupha Wongrostrai, Araya Chiangkhong, Charin Suwanwong, Anon Khunakorncharatphong

**Affiliations:** 1 Kuakarun Faculty of Nursing, Navamindradhiraj university, Bangkok, Thailand; 2 Behavioral Science Research Institute, Srinakharinwirot University, Bangkok, Thailand; 3 Faculty of Medicine Siriraj Hospital, Mahidol University, Bangkok, Thailand

**Keywords:** Recurrent stroke, Working-age, Adult, Urban, Thailand

## Abstract

**Background:**

Stroke survivors, especially working-age adults, face an increased risk of recurrent stroke within one to five years after the initial occurrence, primarily due to suboptimal risk factor management. This study aims to investigate the contributing factors associated with the risk of recurrent stroke in this demographic.

**Methods:**

This case-control study matched participants with recurrent stroke to those without recurrent stroke by age and gender. Multivariate logistic regression analyses were conducted to calculate odds ratios (ORs) with 95% confidence intervals (CIs) to identify significant factors associated with recurrent stroke. The study included 100 patients with recurrent stroke and 200 control participants recruited from the hospital database.

**Results:**

Significant factors associated with recurrent stroke were gender (OR, 1.83; 95% CI, 1.10 to 3.29), high fasting blood sugar (OR, 3.70; 95% CI, 1.10 to 3.29), drinking status (OR, 3.63; 95% CI, 3.01 to 6.54), sedentary lifestyle (OR, 2.77; 95% CI, 1.50 to 5.13), and lack of workplace support for health (OR, 2.02; 95% CI, 1.13 to 3.63). The association of female gender, marital status, smoking status, sedentary lifestyle, interpersonal relationships at the workplace, and workplace support for health with recurrent stroke differed by age group.

**Conclusions:**

This study highlights the importance of addressing lifestyle-related and occupational factors to reduce recurrent stroke risk among working-age adults. Tailoring age-specific stroke prevention strategies, promoting healthier lifestyles, and implementing evidence-based interventions can lead to improved stroke outcomes and enhance the quality of life for this vulnerable population.

## Introduction

Stroke poses a significant global public health challenge, ranking as the second leading cause of death and a substantial contributor to disability. ^1^ Urban centers, such as Bangkok, have witnessed a surge in stroke cases attributed to shifts in socioeconomic factors and the environment. ^2, 3^ Thailand’s urban areas, significantly affected, have undergone lifestyle transformations due to the transition from agrarian to urban-centric living. ^4, 5^ Bangkok, as a prominent example, exemplifies the urban challenges associated with strokes amid rapid urbanization. This urbanization presents both health benefits, like improved medical access, and risks, such as air pollution, the urban heat-island effect, and heightened stress. ^6^

Recent research has drawn attention to a concerning stroke recurrence rate of 53.6% within a year in Thailand, ^7^ shedding light on the risks following an initial stroke. Recurrent strokes result from various health determinants, including uncontrolled factors like alcohol consumption, diabetes, and hypertension, ultimately leading to increased mortality, hospital readmissions, and extended disabilities. ^8– 11^ While age remains a significant predictor of stroke recurrence, vulnerability extends beyond older adults to working-age individuals in urban areas like Bangkok. ^12– 14^ Although returning to work after a stroke can yield positive outcomes, such as improved well-being, ^15, 16^ urban workplaces contribute to recurrence risks due to stress and unhealthy habits.

Despite studies on lifestyle and clinical risk factors for recurrent strokes in working-age adults, ^14, 17^ a research gap still exists. In particular, urban work-related stressors remain unexplored for this demographic. Therefore, this study aims to identify significant factors associated with recurrent stroke in working-age adults in urban areas of Thailand. Gaining insights into these factors will enable the development of targeted interventions and preventive strategies to reduce the risk of recurrent stroke and enhance long-term outcomes and quality of life for this vulnerable population.

## Methods

### Ethical considerations

The study obtained approval from the Institutional Review Board (IRB) of the Faculty of Medicine Vajira Hospital, *Bangkok, Thailand (Approval* no. 110/64 E). Before participation, all participants were informed about the study and their voluntary participation. All collected data was kept confidential and anonymous. Data collection began on September 2021, and continued until March 2022.

### Setting and sample

This case-control study included consecutive patients aged 20-60 years, residing in the urban area of Bangkok for at least 5 years, who experienced their first-ever stroke. Recurrent stroke was confirmed through computerized tomography (CT) or magnetic resonance imaging (MRI) by a physician at the Faculty of Medicine Vajira Hospital in Bangkok, Thailand, between July 2020 to August 2021. Recurrent stroke was defined as a focal neurological deficit lasting more than 24 hours and occurring after the initial stroke. ^18^ Controls were stroke survivors without stroke recurrence, randomly selected from the neurological clinic of a public hospital. Exclusion criteria included patients who were currently unemployed, were unconscious at the time of the stroke, or had any severe complications.

**Figure 1.**
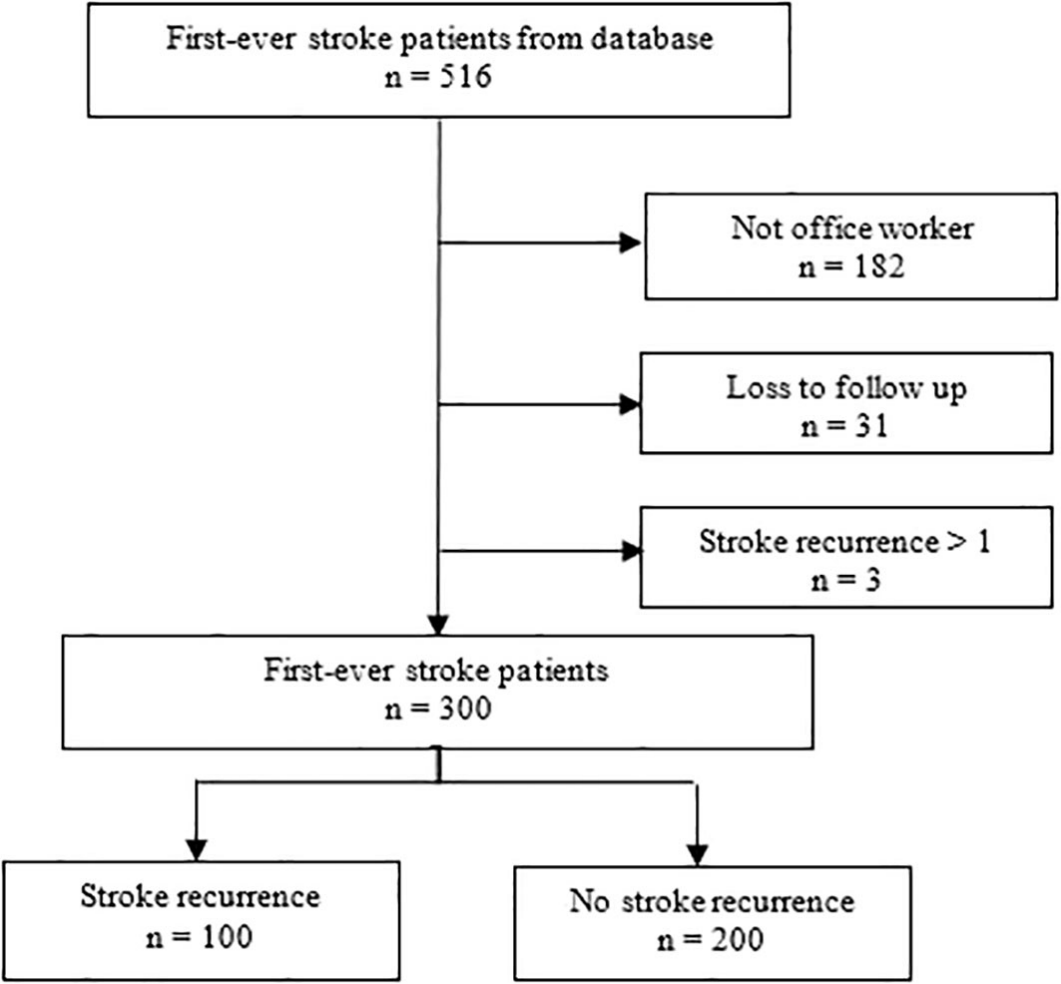
Flowchart of data collection process.

Sample size determination was performed using Epi Info version 7.2.5.0, which is available for free download from the CDC Epi Info website ( https://www.cdc.gov/epiinfo/index.html). The calculation employed a double population formula suitable for an unmatched case-control study. This calculation considered the recurrent stroke rate among controls, which was 50.5%, and an adjusted odds ratio (aOR) of 0.44 derived from a prior study conducted in India. ^19^ To achieve a 95% confidence interval (CI) with 80% statistical power and maintain a controls-to-cases ratio of 2:1, the initial sample size was established at 250 participants. To account for potential non-responses, a buffer of 20% was added to the sample size calculation. Consequently, the total sample size was determined to be 300 participants, containing 100 cases and 200 controls. It’s important to note that out of the 516 first-ever stroke patients in the hospital database, this study specifically included 300 stroke patients. The recruitment process is detailed in Figure 1.

### Instruments

In this study, participants were interviewed by researchers using a structured questionnaire developed based on a literature review of stroke prevention guidelines. ^20^ The questionnaire covered demographic characteristics (age, gender, and marital status), health-related behaviors (preventive health behavior, smoking status, drinking status, and sedentary lifestyle), and occupational factors (interpersonal relationships at the workplace, job characteristics, and physical work environment). Clinical characteristics, such as stroke subtypes, fasting blood sugar (FBS), body mass index (BMI), hypertension, diabetes mellitus, and dyslipidemia, were obtained from medical records. Health-related behaviors, reflecting the past year, were assessed following key guidelines and insights from the literature. Health-related behaviors, gathered retrospectively over the past year, were assessed following the key recommendations and insights from the literature and were organized into four domains: (i) preventive health behavior, (ii) smoking status, (iii) drinking status, and (iv) sedentary lifestyle. Preventive health behavior was measured by compliance with recommended preventive measures, such as medication adherence, physical activity, regular physical examinations, sufficient sleep, maintaining a healthy weight, and a healthy diet (13 items). Participants rated their responses on a 3-point Likert scale ranging from ‘always’ to ‘never’. The Cronbach’s alpha for preventive health behavior was 0.79. Smoking status was determined by asking participants if they currently smoked. The response categories were ‘never’, ‘ever’, and ‘yes’. Participants were classified as non-smokers (never and ever combined) and current smokers. Drinking status was assessed by asking participants if they currently drank alcohol. The response categories were ‘never’, ‘ever’, and ‘yes’. Participants were classified as non-drinkers (never and ever combined) and current drinkers. The sedentary lifestyle was assessed using a 6-item scale specifically developed for this study, which was informed by the definitions of sedentary behavior outlined by Tremblay et al. ^21^ This scale includes activities such as lying down, reclining, and sitting. Participants responded to the items using a 4-point Likert scale ranging from ‘always’ to ‘never.’ The internal consistency of the sedentary lifestyle scale was measured, yielding a Cronbach’s alpha of 0.70.The measurement tool for occupational characteristics was developed based on a thorough review of the relevant literature, which identified three primary domains for assessment: interpersonal relationships in the workplace, job characteristics, and the physical work environment. Interpersonal relationships were evaluated by examining the quality of interactions with colleagues and supervisors, ^22, 23^ employing five specific items that pertain to job autonomy, job feedback, task significance, task identity, and skill variety. Participants rated their experiences using a 3-point Likert scale, ranging from ‘always’ to ‘never.’ The physical work environment was defined according to participants’ perceptions of several factors, including lighting, noise, temperature, and workplace support for health, assessed through four items. Responses were categorized as ‘no’ or ‘yes.’ The Cronbach’s alpha for job characteristics was determined to be 0.79, indicating acceptable internal consistency. Clinical characteristics data were collected from medical records. Stroke subtypes were classified into categories such as embolic, thrombotic, lacunar, and uncertain. ^24^ Current fasting blood sugar (FBS) levels were measured using an oxidase enzymatic method, and body mass index (BMI) was calculated as weight in kilograms divided by height in meters squared. Additionally, historical FBS and BMI data from 6 to 12 months prior were collected for comparison. Hypertension, diabetes mellitus, and dyslipidemia were defined based on physician diagnoses.

### Data collection procedure

Data collection relied on a structure questionnaire administered by trained interviewers. The questionnaire was initially developed in English and then translated into Thai. To ensure accuracy and consistency, it underwent back-translation into English, supervised by a qualified translator.

The questionnaire consisted of both open and closed-ended questions, covering various domains such as demographic characteristics, health-related behavior, and occupational factors. In contrast, clinical data were directly extracted from medical records.

Before commencing full-scale data collection, a pretest was conducted to assess the questionnaire’s reliability and effectiveness, with necessary adjustments made based on the pretest feedback.

To ensure the quality of data collection, both the data collectors and their supervisors underwent comprehensive training. This training included familiarization with the questionnaire, mastery of data collection techniques, understanding of ethical considerations, and a clear grasp of the study’s objectives.

Subsequently, the collected data underwent a rigorous review by the principal investigators to confirm the presence of all necessary information and ensure overall consistency.

### Data analysis

Data were analyzed using STATA 17 software (Serial number: 501706420821).for univariate, bivariate, and multivariate analyses. The chi-square test compared the distribution of all variables in the univariate analysis. Univariate odd ratios (ORs) were calculated for factors with a significant difference ( *p-value* < 0.2). ^25^ Factors meeting this criterion were included in the multivariate analysis, which used logistic regression to calculate adjusted odds ratios (aORs) and 95% confidence interval (CIs) to identify significant factors associated with recurrent stroke. A *p*- *value* < 0.05 indicated statistical significance.

## Results

The study involved 100 cases and 200 controls. Among the cases, 66.0% were aged 25-49 years, compared to 59.5% among controls. Moreover, 58.0% of the cases were male, while the percentage among controls was 65.0%. Most participants were married, with 64.0% in the cases and 57.0% in the controls. Significant differences were observed between cases and controls in various variables, including gender, marital status, FBS, BMI, hypertension, diabetes mellitus, dyslipidemia, smoking status, drinking status, sedentary lifestyle, interpersonal relationships at the workplace, and workplace support for health. Table 1 presents the baseline characteristics of the study participants.

**Table 1.** Baseline characteristics of the study participants (n=300).

Characteristics	Case (n = 100)	Control (n = 200)	*p-value *
	n (%)	n (%)	
Age			.28
25-40 years	66 (66.0)	119 (59.5)	
41-60 years	34 (34.0)	81 (40.5)	
Gender			<.05
Male	54 (54.0)	132 (66.0)	
Female	46 (46.0)	68 (34.0)	
Marital status			<.01
Single	18 (18.0)	70 (35.0)	
Married	64 (64.0)	114 (57.0)	
Widowed/Divorced	18 (18.0)	16 (8.0)	
Fasting Blood Sugar (FBS)			<.01
Normal	21 (21.4)	59 (29.5)	
Medium	34 (34.7)	100 (50.0)	
Hight	43 (43.9)	41 (20.5)	
Body Mass Index (BMI)			<.01
Normal weight	33 (33.0)	107 (53.5)	
Overweight	67 (67.0)	93 (46.5)	
Hypertension			<.05
Yes	67 (67.0)	109 (54.5)	
No	33 (33.0)	91 (45.5)	
Diabetes mellitus			<.05
Yes	50 (50.0)	74 (37.0)	
No	50 (50.0)	126 (63.0)	
Dyslipidemia			<.05
Yes	61 (61.0)	94 (47.0)	
No	39 (39.0)	106 (53.0)	
Smoking status			<.05
Current smoker	47 (47.0)	69 (34.5)	
Non-smoker	53 (53.0)	131 (65.5)	
Drinking status			<.01
Current drinker	55 (55.0)	54 (27.0)	
Non-drinker	45 (45.0)	146 (73.0)	
Preventive health behavior			.51
Low	42 (42.0)	92 (46.0)	
High	58 (58.0)	108 (54.0)	
Sedentary lifestyle			<.01
Low	46 (46.0)	152 (76.0)	
High	54 (54.0)	48 (24.0)	
Interpersonal relationship at workplace			<.01
Low	51 (51.0)	67 (33.5)	
High	49 (49.0)	133 (66.5)	
Job characteristics			.17
Low	42 (42.0)	99 (49.5)	
High	58 (58.0)	101 (50.5)	
Physical work environment			
Light			.57
Enough	50 (50.0)	93 (46.5)	
Poor/too much	50 (50.0)	107 (53.5)	
Noise			.86
Yes	33 (33.0)	68 (34.0)	
No	67 (67.0)	132 (66.0)	
Heat and cold stress			.46
Yes	24 (24.0)	56 (28.0)	
No	76 (76.0)	144 (72.0)	
Workplace support for health			<.01
Yes	54 (54.0)	74 (37.0)	
No	46 (46.0)	126 (63.0)	

^a^ chi-squared for proportions.

Based on the significant differences in the baseline characteristics, specific variables were selected for inclusion in the multivariate analysis model. The results, presented in Table 2, showed that female gender (aOR = 1.83, 95% CI: 1.01, 3.29), high FBS (aOR = 3.70, 95% CI: 1.66, 8.27), drinking status (aOR = 3.63, 95% CI: 2.01, 6.54), sedentary lifestyle (aOR = 2.77, 95% CI: 1.50, 5.13), and lack of workplace support for health (aOR = 2.02, 95% CI: 1.13, 3.63) were significantly associated with recurrent stroke among the working-age adults.

**Table 2.** Logistic regression of predictors for recurrent stroke (n=300).

Predictors	Crude OR (95% CI)	*p*-value	Adjusted OR (95% CI)	*p*-value
Gender (vs. Male)	1.65 (1.01, 2.70)	<.05	1.83 (1.01, 3.29)	<.05
Marital status (vs. Single)				.09
Married	2.18 (1.20, 3.98)	<.05	1.65 (0.81, 3.36)	.17
Widowed/Divorced	4.38 (1.87, 10.23)	<.01	3.13 (1.09, 8.94)	<.05
Fasting Blood Sugar (vs: Normal)				<.01
Medium	0.96 (0.51, 1.80)	.89	1.05 (0.50, 2.18)	.90
High	3.08 (1.60, 5.93)	<.01	3.70 (1.66, 8.27)	<.01
Body Mass Index (BMI) (vs. Low)	2.34 (1.42, 3.85)	<.01	1.76 (0.96, 3.21)	.07
Hypertension (vs. No)	1.70 (1.03, 2.80)	<.05	1.83 (0.99, 3.37)	.05
Diabetes mellitus (vs. No)	1.70 (1.05, 2.77)	<.05	1.38 (0.76, 2.50)	.29
Dyslipidemia (vs. No)	1.76 (1.08, 2.87)	<.05	0.80 (0.43, 1.49)	.49
Smoking status (vs. Non-smoker)	1.68 (1.03, 2.75)	<.01	1.57 (0.87, 2.85)	.14
Drinking status (vs. Non-drinker)	3.30 (2.00, 5.46)	<.01	3.63 (2.01, 6.54)	<.01
Sedentary Lifestyle (vs. Low)	2.70 (1.62, 4.49)	<.01	2.77 (1.50, 5.13)	<.01
Interpersonal relationship at workplace (vs. Low)	0.48 (0.30, 0.79)	<.01	0.56 (0.31, 1.01)	.05
Workplace support for health (vs. Yes)	2.00 (1.23, 3.25)	<.01	2.02 (1.13, 3.63)	<.05

Various factors contributed to recurrent stroke were analyzed into two age groups: individuals aged 25-40 years (“younger workers”) and those aged 41-60 years (“older workers”) ( Table 3). Among younger workers, the most significant factors influencing recurrent stroke were being widowed/divorced (aOR = 7.62, 95% CI: 1.87, 31.12), drinking status (aOR = 4.28, 95% CI: 1.87, 9.80), sedentary lifestyle (aOR = 4.27, 95% CI: 1.84, 9.88), high FBS (aOR = 4.10, 95% CI: 1.32, 12.77), female gender (aOR = 3.28, 95% CI: 1.49, 7.25), being married (aOR = 2.59, 95% CI: 1.02, 6.55), and lack of workplace support for health (aOR = 2.35, 95% CI: 1.05, 5.23). Interestingly, having high interpersonal relationships at the workplace appeared to have a protective effect against recurrent stroke (aOR = 0.34, 95% CI: 0.15, 0.76). On the other hand, among older workers, only drinking status was found to be associated with recurrent stroke (aOR = 3.38, 95% CI: 1.17, 9.72).

**Table 3.** Logistic regression of predictors for recurrent stroke between aged 25-40 years and 41-60 years.

Predictors	Aged 25-40 year (n = 185)				Aged 41-60 year (n = 115)			
	Crude OR (95% CI)	*p-value *	Adjusted OR (95% CI)	*p-value *	Crude OR (95% CI)	*p-value *	Adjusted OR (95% CI)	*p-value *
Gender (vs. Male)	2.89 (1.55, 5.39)	<.01	3.28 (1.49, 7.25)	<.01	0.52 (0.21, 1.30)	.16	0.45 (0.14, 1.50)	.19
**Marital status (vs. Single)**				<.01				.83
Married	3.44 (1.60, 7.40)	<.01	2.59 (1.02, 6.55)	<.05	0.96 (0.35, 2.64)	.94	0.71 (0.20, 2.56)	.60
Widowed/Divorced	9.20 (3.14, 26.98)	<.01	7.62 (1.87, 31.12)	<.01	0.86 (0.17, 4.23)	.85	0.55 (0.06, 5.00)	.60
**Fasting blood sugar (vs: Normal)**				<.05				<.05
Medium	0.88 (0.41, 1.88)	.74	1.60 (0.62, 4.17)	.33	0.99 (0.31, 3.20)	.99	0.76 (0.17, 3.41)	.72
High	2.62 (1.13, 6.08)	<.05	4.10 (1.32, 12.77)	<.05	4.33 (1.47, 12.79)	<.01	4.07 (0.83, 19.98)	.08
Body mass index (BMI) (vs. Low)	2.25 (1.21, 4.19)	<.05	1.95 (0.85, 4.44)	.11	2.71 (1.13, 6.52)	<.05	1.53 (0.51, 4.58)	.45
Hypertension (vs. No)	1.47 (0.80, 2.70)	.22	1.87 (0.84, 4.15)	.12	2.89 (1.08, 7.78)	<.05	2.44 (0.68, 8.79)	.16
Diabetes mellitus (vs. No)	1.61 (0.88, 2.96)	.12	1.25 (0.56, 2.81)	.59	1.77 (0.78, 4.04)	.18	2.08 (0.64, 6.77)	.22
Dyslipidemia (vs. No)	1.44 (0.78, 2.63)	.24	0.53 (0.23, 1.23)	.13	2.72 (1.15, 6.40)	<.05	1.08 (0.28, 4.18)	.91
Smoking status (vs. Non-smoker)	1.21 (0.66, 2.24)	.54	1.15 (0.50, 2.60)	.75	3.01 (1.31, 6.89)	<.01	2.75 (0.99, 7.62)	.05
Drinking status (vs. Non-drinker)	2.83 (1.52, 5.27)	<.01	4.28 (1.87, 9.80)	<.01	4.24 (1.79, 10.01)	<.01	3.38 (1.17, 9.72)	<.05
Sedentary lifestyle (vs. Low)	3.41 (1.81, 6.42)	<.01	4.27 (1.84, 9.88)	<.01	1.57 (0.63, 3.90)	.33	1.57 (0.43, 5.80)	.50
Interpersonal relationships at the workplace (vs. Low)	0.45 (0.24, 0.84)	<.05	0.34 (0.15, 0.76)	<.01	0.48 (0.21, 1.09)	.08	0.96 (0.33, 2.80)	.94
Workplace support for health (vs. non-support)	2.32 (1.25, 4.29)	<.01	2.35 (1.05, 5.23)	<.05	1.66 (0.74, 3.73)	.22	2.47 (0.81, 7.53)	.11

## Discussion

Patients who have experienced their first-ever stroke remain at high risk of recurrent stroke, particularly in Bangkok, Thailand, due to lifestyle-related risk factors and occupational factors among working-age adults. This study aimed to identify significant factors associated with recurrent stroke in this population. The results revealed that female gender, high FBS, drinking status, sedentary lifestyle, and lack of workplace support for health were significant factors for recurrent stroke. Moreover, there were differences in the pattern of significant factors between younger workers and older workers.

The study identified female gender as a significant factor for recurrent stroke among working-age adults, indicating that women in this age group are at higher risk of recurrent strokes compared to men. The impact of gender on stroke risk may vary depending on age groups. The study revealed a stronger association between female gender and recurrent stroke in young workers, while no significant association was observed in older workers. The difference may be attributed to lifestyle factors. Younger women living in urban area may be more vulnerable to specific risk factors related to recurrent stroke, such as lifestyle choices, exposure to air pollution, or work-related stress. ^25– 29^ To gain a deeper understanding of the mechanisms driving these gender-specific differences in recurrent stroke risk, further research is warranted. Exploring the interactions between age, gender, and other risk factors could offer valuable insights for tailoring effective stroke prevention strategies to specific populations.

Marital status significantly influences recurrent stroke in working-age adults, particularly among younger workers. Married or widowed/divorced women in this age group faced a higher risk of recurrent strokes compared to single women. However, no significant association between marital status and recurrent stroke was observed in older workers. This age-specific disparity in the relationship between marital status and recurrent stroke has important implications. For younger workers, being married or experiencing a change in marital status could potentially be associated with increased stress, lifestyle changes, or other factors contributing to a higher stroke risk. ^30– 34^ Further research is needed to better understand the underlying reasons behind these age-specific associations between marital status and recurrent stroke risk.

Fasting blood sugar (FBS) showed a significant association with an increased risk of recurrent stroke among younger workers. Previous studies have indicated that individuals without a history of diabetes mellitus but with fasting blood sugar level >7mmol/L, or those with prediabetes, are considered at risk for developing recurrent stroke. ^35– 38^ However, in our study, no significant association between FBS and recurrent stroke was observed in older workers. This finding suggests that FBS levels may not play a prominent role as a risk factor for recurrent stroke in the older age group, where other factors may have a more significant impact on stroke risk. Understanding these age-specific differences in this association is crucial for stroke management and prevention strategies. For younger workers, early detection and management of elevated FBS levels may be critical in mitigating the risk of recurrent stroke.

Our findings revealed that current drinkers, both among younger and older workers, had a higher risk of recurrent stroke compared to non-drinkers. While previous research has connected alcohol consumption to stroke recurrence in elderly patients, ^8^ its association with recurrent stroke in working-age adults is less clear. However, studies have suggested that heavy alcohol consumption is a modifiable risk factor for stroke in this age group. ^39– 42^ Addressing alcohol consumption is crucial in stroke prevention strategies for working-age adults. Healthcare providers should educate patients about the potential risks and promote healthier lifestyle choices. Understanding the specific patterns of alcohol consumption in different age groups can help tailor effective intervention programs. Reducing heavy alcohol intake and promoting responsible drinking behaviors may significantly lower the burden of recurrent stroke among working-age adults.

Our study found a significant association between sedentary lifestyle and increased recurrent stroke risk among younger workers. One plausible explanation includes younger urban workers spending more time in sedentary activities due to work or lifestyle choices, ^43– 46^ contributing to the elevated risk of recurrent stroke. However, no significant association between sedentary lifestyle and recurrent stroke was observed in older workers, where diverse physical activity levels and other factors may play a more prominent role in influencing risk. Interestingly, Vilhelmson et al. ^47^ showed that younger workers allocate more time to work and digital leisure, while older workers engage more in outdoor activity and exercise. Understanding these age-specific associations is essential for designing targeted stroke prevention strategies. For younger workers, interventions to reduce sedentary behavior and promoting regular physical activity may be crucial in mitigating recurrent stroke risk. Implementing workplace wellness programs and encouraging regular breaks from sedentary activities can be beneficial.

Our study identified a significant association between lack of workplace support for health and an increased risk of recurrent stroke among younger workers. Interestingly, workplace support seems to have no such impact on stroke risk for older workers. The key lies in how workplace support may indirectly influence lifestyle behaviors among younger individuals. Young workers may face extended working hours, fostering high-stress and competitive environments as they strive to establish their careers. This can lead to overworking and result in unhealthy habits such as poor diet, reduced physical activity, and increased alcohol intake – all contributing to stroke risks factors like obesity, hypertension, and diabetes. ^48– 50^ Moreover, the stress from this work culture can lead to insufficient sleep and chronic stress, both of which are linked to an increased risk of stroke. ^51, 52^ Young workers, prioritizing productivity, may disregard health warnings, further enhancing their risk. Conversely, older workers tend to have better psychological health compared to younger workers. ^53^ They are more likely to seek medical help when needed and adhere to healthier behaviors, thus mitigating the stroke risks that younger workers may face. Understanding the differential impact of workplace support on stroke risk in different age groups is essential for developing targeted prevention strategies. For younger workers, interventions focusing on promoting healthier lifestyles and addressing the impact of work-related stress could be crucial in reducing their risk of recurrent stroke.

Our study revealed a significant association between workplace interpersonal relationships and recurrent stroke among working-age adults. Interestingly, we found that these relationships acted as a significant protective factor against recurrent stroke among younger workers but did not seem to have the same impact on stroke risk for older workers. Positive interpersonal relationships in the workplace may play a crucial role in buffering against the risk of recurrent stroke among younger workers, contributing to reduced stress levels, improved mental well-being, and overall job satisfaction. ^54, 55^ As younger workers often face the challenges of establishing themselves in their careers and navigating work-related stress, a positive social environment at work can act as a protective factor, influencing their health outcomes, including stroke risk. ^56^ In contrast, older workers may have developed better coping mechanisms and emotional resilience, which could mitigate the impact of workplace relationships on their adverse health outcomes and promote well-being. ^57^ Understanding the differential influence of workplace interpersonal relationships on stroke risk in different age group is vital for designing targeted interventions. Reducing work-related stress could be a valuable component of stroke prevention strategies, particularly for younger workers.

Examining these findings through the socio-ecological model ^58^ provides deeper insight into the intricate factors influencing urban areas. Individual behaviors like drinking habits and physical activity levels intersect with the broader urban landscape, including workplace dynamics and healthcare services accessibility. These interactions highlight the necessity for comprehensive, multi-level interventions that address both individual choices and the urban milieu. Effort to mitigate recurrent stroke risk among urban working-age adults should not solely concentrate on individual risk factors but should also consider the urban environment holistically. This involves promoting healthier behaviors, enhancing workplace health support, and improving healthcare services accessibility within the city. By aligning interventions with socio-ecological model principles, healthcare providers and policymakers can collaboratively craft a healthier urban environment, consequently alleviating the burden of recurrent stroke on this vulnerable population.

Several limitations of this study may influence the interpretation of the results. First, this study’s single-site hospital setting may limit the generalizability of the findings. While the study provides valuable insights into the specific urban setting, caution should be exercised when extrapolating these results to different geographic locations or diverse healthcare settings. To enhance external validity, future research should include larger and more diverse samples from multiple healthcare centers or regions. Second, a notable limitation lies in the absence of detailed information on medication use, physical activity, work-related stress, and the urban environment relevant to working-age adults. These factors can play crucial roles in influencing stroke risk and recurrence among this population. Collecting comprehensive data on these variables in future research would lead to a more profound understanding of their contributions to recurrent stroke risk, thereby guiding better-informed stroke prevention strategies. Lastly, it is essential to recognize that the risk factors examined in this study may interact with one another, leading to mutual influences and complex interplay. Comorbidities and the interactions between risk factors can significantly affect stroke recurrence risk, warranting thorough investigation. Future studies should aim to explore the synergistic effects of multiple risk factors and consider potential confounding variables to elucidate a more comprehensive picture of stroke recurrence risk among working-age adults.

## Conclusion

In conclusion, this study sheds light on the factors associated with recurrent stroke among working-age adults in urban areas of Bangkok, Thailand. Addressing lifestyle-related and occupational risk factors is crucial for reducing recurrent stroke risk in this vulnerable population. The impact of these factors varied across different age groups, necessitating age-specific stroke prevention strategies. Promoting healthier lifestyle, reducing alcohol consumption, encouraging physical activity, and addressing work-related stress are vital steps in mitigating recurrent stroke risk among working-age adults. Healthcare providers can develop targeted and effective stroke prevention and management strategies, including healthy lifestyle promotion, alcohol reduction, physical activity encouragement, stress management, regular health check-ups, multidisciplinary care, and workplace wellness initiatives. Implementing evidence-based interventions can lead to improved stroke outcomes, reduced disability, and an enhanced quality of life for urban working-age adults.

## Declarations

### Ethical considerations

The study obtained approval from the Institutional Review Board (IRB) of the Faculty of Medicine Vajira Hospital, *Bangkok, Thailand (Approval* no. 110/64 E). The approval was granted on August 2, 2021, and is valid until August 1, 2022.

*Consent statement*

Before participation, all participants were informed about the study and their right to voluntary participation. They provided written informed consent prior to being enrolled in the study. All collected data was kept confidential and anonymous, ensuring the privacy of all participants. This study adhered to the principles outlined in the Declaration of Helsinki, ensuring ethical conduct.

## Author contribution statement

All author listed have contributed to the development and the writing of this article.

## Additional information

No additional information is available for this paper.

## Data Availability

*Ethical and security consideration* The data consists of personal medical records of patients, and access is restricted to protect patient confidentiality. To apply for access to the data, readers or reviewers must submit a formal request including the purpose of the data use, a detailed research plan, and proof of ethical approval from a recognized institutional review board (IRB). Access will be granted only under the condition that the data will be used solely for the approved research purposes, and all necessary measures to ensure data privacy and security are in place. Applications should be directed to the corresponding author, and each request will be reviewed on a case-by-case basis.
