# Lifestyle and Occupational Factors Associated with Recurrent Stroke among Working-Age Adults in Urban Areas of Thailand

**DOI:** 10.12688/f1000research.154968.2

**Published:** 2025-05-23

**Authors:** Yupha Wongrostrai, Araya Chiangkhong, Charin Suwanwong, Anon Khunakorncharatphong

**Affiliations:** 1 Kuakarun Faculty of Nursing, Navamindradhiraj university, Bangkok, Thailand; 2 Behavioral Science Research Institute, Srinakharinwirot University, Bangkok, Thailand; 3 Faculty of Medicine Siriraj Hospital, Mahidol University, Bangkok, Thailand

**Keywords:** Recurrent stroke, Working-age, Adult, Urban, Thailand

## Abstract

**Background:**

Stroke survivors, particularly those of working age, are at an increased risk of recurrent stroke within one–five years of the initial event, largely due to suboptimal management of risk factors. This study aimed to identify lifestyle and occupational factors associated with recurrent stroke in this demographic population.

**Methods:**

This case-control study included 100 patients with recurrent ischemic stroke and 200 ischemic stroke survivors without recurrence, who were recruited from the hospital database. Multivariate logistic regression was used to identify significant factors associated with recurrence, which were presented as adjusted odds ratios (aORs) with 95% confidence intervals (CIs).

**Results:**

The mean age was 45.4 years (SD = 15.1) among cases and 50.6 years (SD = 6.5) among controls. The male-to-female ratios were 1.17:1 and 1.94:1 in the case and control groups, respectively. Significant factors associated with recurrent stroke included female sex (aOR: 1.83; 95% CI [1.10–3.29]), high fasting blood sugar (aOR: 3.70; 95% CI [1.66–8.27]), current alcohol consumption (aOR: 3.63; 95% CI [2.01–6.54]), sedentary lifestyle (aOR: 2.77; 95% CI [1.50–5.13]), and lack of workplace support for health (aOR: 2.02; 95% CI [1.13–3.63]). The associations between these factors and stroke recurrence varied according to the age group.

**Conclusions:**

This study highlights the critical role of modifiable lifestyle and occupational factors in stroke recurrence among working-age adults. Tailored age-specific prevention strategies—emphasizing physical activity, reduced alcohol use, and improved workplace health environments—may reduce the risk of recurrence and enhance health outcomes in this population.

## Introduction

Stroke poses a significant global public health challenge, ranking as the second leading cause of death and a substantial contributor to disability. ^1^ Urban centers, such as Bangkok, have witnessed a surge in stroke cases attributed to shifts in socioeconomic factors and the environment. ^2, 3^ Thailand’s urban areas, significantly affected, have undergone lifestyle transformations due to the transition from agrarian to urban-centric living. ^4, 5^ Bangkok, as a prominent example, exemplifies the urban challenges associated with strokes amid rapid urbanization. This urbanization presents both health benefits, like improved medical access, and risks, such as air pollution, the urban heat-island effect, and heightened stress. ^6^

Recent research has drawn attention to a concerning stroke recurrence rate of 53.6% within a year in Thailand, ^7^ shedding light on the risks following an initial stroke. Recurrent strokes often result from inadequately controlled modifiable risk factors, including excessive alcohol consumption, poorly managed diabetes, and uncontrolled hypertension, ultimately leading to increased mortality, hospital readmissions, and prolonged disability. ^8– 11^ While age remains a significant predictor of stroke recurrence, vulnerability extends beyond older adults to working-age individuals (defined as those aged 18–60 years) in urban areas like Bangkok. ^12– 14^

Although returning to work after a stroke can yield positive outcomes, such as improved well-being, ^15, 16^ urban workplaces contribute to recurrence risks due to stress and unhealthy habits.

Despite studies on lifestyle and clinical risk factors for recurrent strokes in working-age adults, ^14, 17^ a research gap still exists. In particular, urban work-related stressors remain unexplored for this demographic. These stressors include psychosocial and environmental stress factors commonly experienced by individuals working in urban settings, such as high job demands, limited workplace support, interpersonal conflicts, and limited physical activity due to sedentary working conditions. ^18, 19^ Therefore, this study aims to identify significant factors associated with recurrent stroke in working-age adults in urban areas of Thailand. Gaining insights into these factors will enable the development of targeted interventions and preventive strategies to reduce the risk of recurrent stroke and enhance long-term outcomes and quality of life for this vulnerable population.

## Methods

### Setting and sample

This study employed a case-control design. Participants were classified based on their recurrent stroke status. This study included consecutive working-age adults (aged 20–60 years) who were diagnosed with any type of stroke at the Faculty of Medicine Vajira Hospital, Bangkok, Thailand, between July 2020 and August 2021. Cases were defined as patients who had experienced a recurrent stroke, which was confirmed by a neurologist using computerized tomography (CT) or magnetic resonance imaging (MRI), and characterized by a new focal neurological deficit lasting more than 24 hours following an initial stroke event. ^20^ Controls were stroke survivors without any documented recurrence who attended regular follow-up visits to the hospital’s neurology outpatient department during the same period. Controls were randomly selected from the hospital registry and matched according to inclusion criteria.

Both cases and controls were required to have continuously resided in the urban areas of Bangkok for at least five years prior to participation. Exclusion criteria included unemployment at the time of recruitment, loss of consciousness at stroke onset, severe complications such as hemorrhagic transformation, transient ischemic attack (TIA), or other significant neurological impairments that could affect accurate behavioral recall. Participants were stratified into age groups (25–40 and 41–60 years) based on occupational health literature, which classifies these age bands into early- and mid-to-late-career stages. ^21, 22^

The sample size was calculated using Epi Info software version 7.2.5.0 (CDC, Atlanta, USA), available at: https://www.cdc.gov/epiinfo/index.html.The calculation used a double population formula suitable for an unmatched case-control study, based on a recurrent stroke rate among controls of 50.5%, and an adjusted odds ratio (aOR) of 0.44, derived from a prior study conducted in India. ^23^ To achieve a 95% confidence interval (CI) with 80% statistical power and maintain a controls-to-cases ratio of 2:1. The initial sample size was 250. An additional 20% was added to account for potential non-responses, resulting in a final total of 300 participants (100 cases and 200 controls).

### Instruments

A structured questionnaire was developed based on a literature review of stroke prevention guidelines. ^24^ The tool consists of items covering three main domains: demographic characteristics (age, gender, and marital status), health-related behaviors, and occupational factors. Health-related behaviors were retrospectively assessed over the past year and categorized into four domains: (i) preventive health behavior, (ii) smoking status, (iii) drinking status, and (iv) a sedentary lifestyle.

The questionnaire covered demographic characteristics (age, gender, and marital status), health-related behaviors (preventive health behavior, smoking status, drinking status, and sedentary lifestyle), and occupational factors (interpersonal relationships at the workplace, job characteristics, and physical work environment). Clinical characteristics, such as stroke subtypes, fasting blood sugar (FBS), body mass index (BMI), hypertension, diabetes mellitus, and dyslipidemia, were obtained from medical records. Health-related behaviors, reflecting the past year, were assessed following key guidelines and insights from the literature. Health-related behaviors, gathered retrospectively over the past year, were assessed following the key recommendations and insights from the literature and were organized into four domains: (i) preventive health behavior, (ii) smoking status, (iii) drinking status, and (iv) sedentary lifestyle. Preventive health behavior was measured by compliance with recommended preventive measures, such as medication adherence, physical activity, regular physical examinations, sufficient sleep, maintaining a healthy weight, and a healthy diet (13 items). Participants rated their responses on a 3-point Likert scale ranging from ‘always’ to ‘never’. The Cronbach’s alpha for preventive health behavior was 0.79. Smoking status was determined by asking participants if they currently smoked. The response categories were ‘never’, ‘ever’, and ‘yes’. Participants were classified as non-smokers (never and ever combined) and current smokers. Drinking status was assessed by asking participants if they currently drank alcohol. The response categories were ‘never’, ‘ever’, and ‘yes’. Participants were classified as non-drinkers (never and ever combined) and current drinkers. The sedentary lifestyle was assessed using a 6-item scale specifically developed for this study, which was informed by the definitions of sedentary behavior outlined by Tremblay et al. ^25^ This scale includes activities such as lying down, reclining, and sitting. Participants responded to the items using a 4-point Likert scale ranging from ‘always’ to ‘never.’ The internal consistency of the sedentary lifestyle scale was measured, yielding a Cronbach’s alpha of 0.70.

The measurement tool for occupational characteristics was developed based on a thorough review of the relevant literature, which identified three primary domains for assessment: interpersonal relationships in the workplace, job characteristics, and the physical work environment. Interpersonal relationships were evaluated by examining the quality of interactions with colleagues and supervisors, ^26, 27^ employing five specific items that pertain to job autonomy, job feedback, task significance, task identity, and skill variety. Participants rated their experiences using a 3-point Likert scale, ranging from ‘always’ to ‘never.’ The physical work environment was defined according to participants’ perceptions of several factors, including lighting, noise, temperature, and workplace support for health, assessed through four items. Responses were categorized as ‘no’ or ‘yes.’ The Cronbach’s alpha for job characteristics was determined to be 0.79, indicating acceptable internal consistency. Clinical characteristics data were collected from medical records. Stroke subtypes were classified into categories such as embolic, thrombotic, lacunar, and uncertain. ^28^

Current fasting blood sugar (FBS) levels were measured using an oxidase enzymatic method. FBS was measured in mg/dL using the oxidase enzymatic method. FBS was categorized as: Normal (<100 mg/dL), Medium (100–125 mg/dL), and High (≥126 mg/dL), based on ADA guidelines. Body mass index (BMI) was calculated as weight in kilograms divided by height in meters squared. Additionally, historical FBS and BMI data from 6 to 12 months prior were collected for comparison. Hypertension, diabetes mellitus, and dyslipidemia were defined based on physician diagnoses.

### Data collection procedure

Data were collected through face-to-face interviews conducted by nurses specializing in stroke care, using the structured questionnaire described previously. These nurses underwent comprehensive training encompassing the study objectives, questionnaire content, ethical considerations, and standardized procedures for data collection. All the completed questionnaires were reviewed daily by senior investigators to ensure data accuracy, completeness, and consistency.

The participants provided information regarding their stroke history, date of diagnosis, clinical manifestations, lifestyle-related risk factors, and family history of chronic diseases. Physical examination was conducted to assess height, weight, and blood pressure. Clinical data—including stroke subtypes, FBS, BMI, and comorbid conditions—were extracted directly from medical records.

### Data analysis

Data were analyzed using STATA 17 software (Serial number: 501706420821).for univariate, bivariate, and multivariate analyses. The chi-square test compared the distribution of all variables in the univariate analysis. Univariate odd ratios (ORs) were calculated for factors with a significant difference ( *p-value* < 0.2). ^29^ Factors meeting this criterion were included in the multivariate analysis, which used logistic regression to calculate adjusted odds ratios (aORs) and 95% confidence interval (CIs) to identify significant factors associated with recurrent stroke. A *p*- *value* < 0.05 indicated statistical significance.

## Results

**Figure 1.**
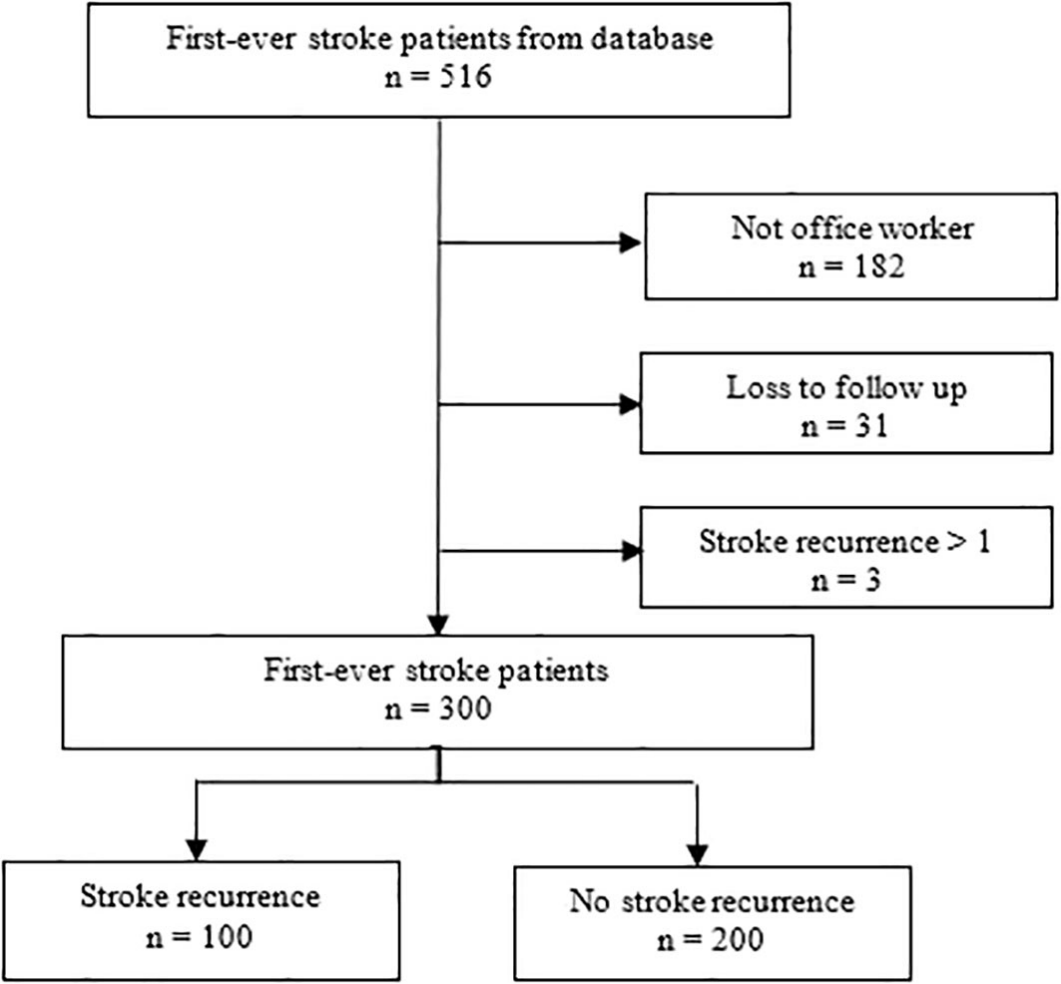
Flowchart of participant selection process (patients with more than one stroke recurrence were excluded).

From a total of 516 eligible first-ever stroke patients initially identified from the hospital database, 300 patients who met all inclusion criteria were included in this study. The mean age was 45.4 years (SD = 15.1) in the case group and 50.6 years (SD = 6.5) in the control group. The male-to-female ratio was 1.17:1 among cases and 1.94:1 among controls. Figure 1 illustrates the detailed recruitment and participant selection processes, showing reasons for exclusion such as non-office workers (n=182), loss to follow-up (n=31), and patients with more than one stroke recurrence (n=3).

A total of 300 participants were included in the study: 100 patients with recurrent stroke and 200 controls without recurrence. The mean age of 43.1 years in the case group and 44.2 years in the control group. In terms of sex distribution, 54.0% of the cases and 66.0% of the controls were male, corresponding to male-to-female ratios of 1.17:1 and 1.94:1, respectively. Most participants were married (64.0% in cases and 57.0% in controls). Statistically significant differences between the two groups were found in sex, marital status, fasting blood sugar (FBS), body mass index (BMI), hypertension, diabetes mellitus, dyslipidemia, smoking, alcohol consumption, sedentary lifestyle, interpersonal relationships at the workplace, and workplace support for health. The baseline characteristics of the participants are summarized in Table 1.

**Table 1.** Baseline characteristics of the study participants (n=300).

Characteristics	Case (n = 100)	Control (n = 200)	*p-value *
	n (%)	n (%)	
Age			.28
25-40 years	66 (66.0)	119 (59.5)	
41-60 years	34 (34.0)	81 (40.5)	
*M*+ *SD*	45.4 ± 15.1	50.6 ± 6.5	
Gender			<.05
Male	54 (54.0)	132 (66.0)	
Female	46 (46.0)	68 (34.0)	
Male-to-Female Ratio	1.94:1	1.17:1	
Marital status			<.01
Single	18 (18.0)	70 (35.0)	
Married	64 (64.0)	114 (57.0)	
Widowed/Divorced	18 (18.0)	16 (8.0)	
Fasting Blood Sugar (FBS)			<.01
Normal	21 (21.4)	59 (29.5)	
Medium	34 (34.7)	100 (50.0)	
Hight	43 (43.9)	41 (20.5)	
Body Mass Index (BMI)			<.01
Normal weight	33 (33.0)	107 (53.5)	
Overweight	67 (67.0)	93 (46.5)	
Hypertension			<.05
Yes	67 (67.0)	109 (54.5)	
No	33 (33.0)	91 (45.5)	
Diabetes mellitus			<.05
Yes	50 (50.0)	74 (37.0)	
No	50 (50.0)	126 (63.0)	
Dyslipidemia			<.05
Yes	61 (61.0)	94 (47.0)	
No	39 (39.0)	106 (53.0)	
Smoking status			<.05
Current smoker	47 (47.0)	69 (34.5)	
Non-smoker	53 (53.0)	131 (65.5)	
Drinking status			<.01
Current drinker	55 (55.0)	54 (27.0)	
Non-drinker	45 (45.0)	146 (73.0)	
Preventive health behavior			.51
Low	42 (42.0)	92 (46.0)	
High	58 (58.0)	108 (54.0)	
Sedentary lifestyle			<.01
Low	46 (46.0)	152 (76.0)	
High	54 (54.0)	48 (24.0)	
Interpersonal relationship at workplace			<.01
Low	51 (51.0)	67 (33.5)	
High	49 (49.0)	133 (66.5)	
Job characteristics			.17
Low	42 (42.0)	99 (49.5)	
High	58 (58.0)	101 (50.5)	
Physical work environment			
Light			.57
Enough	50 (50.0)	93 (46.5)	
Poor/too much	50 (50.0)	107 (53.5)	
Noise			.86
Yes	33 (33.0)	68 (34.0)	
No	67 (67.0)	132 (66.0)	
Heat and cold stress			.46
Yes	24 (24.0)	56 (28.0)	
No	76 (76.0)	144 (72.0)	
Workplace support for health			<.01
Yes	54 (54.0)	74 (37.0)	
No	46 (46.0)	126 (63.0)	

^a^ chi-squared for proportions.

Based on the significant differences in the baseline characteristics, specific variables were selected for inclusion in the multivariate analysis model. The results, presented in Table 2, showed that female gender (aOR = 1.83, 95% CI: 1.01, 3.29), high FBS (aOR = 3.70, 95% CI: 1.66, 8.27), drinking status (aOR = 3.63, 95% CI: 2.01, 6.54), sedentary lifestyle (aOR = 2.77, 95% CI: 1.50, 5.13), and lack of workplace support for health (aOR = 2.02, 95% CI: 1.13, 3.63) were significantly associated with recurrent stroke among the working-age adults.

**Table 2.** Logistic regression of predictors for recurrent stroke (n=300).

Predictors	Crude OR (95% CI)	*p*-value	Adjusted OR (95% CI) *	*p*-value
Gender (vs. Male)	1.65 (1.01, 2.70)	<.05	1.83 (1.01, 3.29)	<.05
Marital status (vs. Single)				.09
Married	2.18 (1.20, 3.98)	<.05	1.65 (0.81, 3.36)	.17
Widowed/Divorced	4.38 (1.87, 10.23)	<.01	3.13 (1.09, 8.94)	<.05
Fasting Blood Sugar (vs: Normal)				<.01
Medium	0.96 (0.51, 1.80)	.89	1.05 (0.50, 2.18)	.90
High	3.08 (1.60, 5.93)	<.01	3.70 (1.66, 8.27)	<.01
Body Mass Index (BMI) (vs. Low)	2.34 (1.42, 3.85)	<.01	1.76 (0.96, 3.21)	.07
Hypertension (vs. No)	1.70 (1.03, 2.80)	<.05	1.83 (0.99, 3.37)	.05
Diabetes mellitus (vs. No)	1.70 (1.05, 2.77)	<.05	1.38 (0.76, 2.50)	.29
Dyslipidemia (vs. No)	1.76 (1.08, 2.87)	<.05	0.80 (0.43, 1.49)	.49
Smoking status (vs. Non-smoker)	1.68 (1.03, 2.75)	<.01	1.57 (0.87, 2.85)	.14
Drinking status (vs. Non-drinker)	3.30 (2.00, 5.46)	<.01	3.63 (2.01, 6.54)	<.01
Sedentary Lifestyle (vs. Low)	2.70 (1.62, 4.49)	<.01	2.77 (1.50, 5.13)	<.01
Interpersonal relationship at workplace (vs. Low)	0.48 (0.30, 0.79)	<.01	0.56 (0.31, 1.01)	.05
Workplace support for health (vs. Yes)	2.00 (1.23, 3.25)	<.01	2.02 (1.13, 3.63)	<.05

* Adjusted for gender, marital status, FBS, BMI, hypertension, diabetes mellitus, dyslipidemia, smoking, drinking, sedentary lifestyle, interpersonal relationships at the workplace, and workplace support for health.

Various factors contributed to recurrent stroke were analyzed into two age groups: individuals aged 25-40 years (early-career workers) and those aged 41-60 years (mid-to-late-career workers) ( Table 3). Among early-career workers, the most significant factors influencing recurrent stroke were being widowed/divorced (aOR = 7.62, 95% CI: 1.87, 31.12), drinking status (aOR = 4.28, 95% CI: 1.87, 9.80), sedentary lifestyle (aOR = 4.27, 95% CI: 1.84, 9.88), high FBS (aOR = 4.10, 95% CI: 1.32, 12.77), female gender (aOR = 3.28, 95% CI: 1.49, 7.25), being married (aOR = 2.59, 95% CI: 1.02, 6.55), and lack of workplace support for health (aOR = 2.35, 95% CI: 1.05, 5.23). Interestingly, having high interpersonal relationships at the workplace appeared to have a protective effect against recurrent stroke (aOR = 0.34, 95% CI: 0.15, 0.76). On the other hand, among mid-to-late-career workers, only drinking status was found to be associated with recurrent stroke (aOR = 3.38, 95% CI: 1.17, 9.72).

**Table 3.** Logistic regression of predictors for recurrent stroke between aged 25-40 years and 41-60 years.

Predictors	Aged 25-40 year (n = 185)				Aged 41-60 year (n = 115)			
	Crude OR (95% CI)	*p-value *	Adjusted OR (95% CI)	*p-value *	Crude OR (95% CI)	*p-value *	Adjusted OR (95% CI)	*p-value *
Gender (vs. Male)	2.89 (1.55, 5.39)	<.01	3.28 (1.49, 7.25)	<.01	0.52 (0.21, 1.30)	.16	0.45 (0.14, 1.50)	.19
**Marital status (vs. Single)**				<.01				.83
Married	3.44 (1.60, 7.40)	<.01	2.59 (1.02, 6.55)	<.05	0.96 (0.35, 2.64)	.94	0.71 (0.20, 2.56)	.60
Widowed/Divorced	9.20 (3.14, 26.98)	<.01	7.62 (1.87, 31.12)	<.01	0.86 (0.17, 4.23)	.85	0.55 (0.06, 5.00)	.60
**Fasting blood sugar (vs: Normal)**				<.05				<.05
Medium	0.88 (0.41, 1.88)	.74	1.60 (0.62, 4.17)	.33	0.99 (0.31, 3.20)	.99	0.76 (0.17, 3.41)	.72
High	2.62 (1.13, 6.08)	<.05	4.10 (1.32, 12.77)	<.05	4.33 (1.47, 12.79)	<.01	4.07 (0.83, 19.98)	.08
Body mass index (BMI) (vs. Low)	2.25 (1.21, 4.19)	<.05	1.95 (0.85, 4.44)	.11	2.71 (1.13, 6.52)	<.05	1.53 (0.51, 4.58)	.45
Hypertension (vs. No)	1.47 (0.80, 2.70)	.22	1.87 (0.84, 4.15)	.12	2.89 (1.08, 7.78)	<.05	2.44 (0.68, 8.79)	.16
Diabetes mellitus (vs. No)	1.61 (0.88, 2.96)	.12	1.25 (0.56, 2.81)	.59	1.77 (0.78, 4.04)	.18	2.08 (0.64, 6.77)	.22
Dyslipidemia (vs. No)	1.44 (0.78, 2.63)	.24	0.53 (0.23, 1.23)	.13	2.72 (1.15, 6.40)	<.05	1.08 (0.28, 4.18)	.91
Smoking status (vs. Non-smoker)	1.21 (0.66, 2.24)	.54	1.15 (0.50, 2.60)	.75	3.01 (1.31, 6.89)	<.01	2.75 (0.99, 7.62)	.05
Drinking status (vs. Non-drinker)	2.83 (1.52, 5.27)	<.01	4.28 (1.87, 9.80)	<.01	4.24 (1.79, 10.01)	<.01	3.38 (1.17, 9.72)	<.05
Sedentary lifestyle (vs. Low)	3.41 (1.81, 6.42)	<.01	4.27 (1.84, 9.88)	<.01	1.57 (0.63, 3.90)	.33	1.57 (0.43, 5.80)	.50
Interpersonal relationships at the workplace (vs. Low)	0.45 (0.24, 0.84)	<.05	0.34 (0.15, 0.76)	<.01	0.48 (0.21, 1.09)	.08	0.96 (0.33, 2.80)	.94
Workplace support for health (vs. non-support)	2.32 (1.25, 4.29)	<.01	2.35 (1.05, 5.23)	<.05	1.66 (0.74, 3.73)	.22	2.47 (0.81, 7.53)	.11

## Discussion

Patients who have experienced their first-ever stroke remain at high risk of recurrent stroke, particularly in Bangkok, Thailand, due to lifestyle-related risk factors and occupational factors among working-age adults. This study aimed to identify significant factors associated with recurrent stroke in this population. The results revealed that female gender, high FBS, drinking status, sedentary lifestyle, and lack of workplace support for health were significant factors for recurrent stroke. Moreover, there were differences in the pattern of significant factors between early-career workers and mid-to-late-career workers.

The study identified female gender as a significant factor for recurrent stroke among working-age adults, indicating that women in this age group are at higher risk of recurrent strokes compared to men. This association was stronger among early-career workers, while no significant relationship was observed among mid-to-late-career workers. Previous research suggests that younger women living in urban areas may be more vulnerable to specific recurrent stroke risk factors, such as lifestyle choices, exposure to air pollution, or work-related stress. ^29– 33^ The differences observed may thus be attributed to these lifestyle-related factors. To gain a deeper understanding of the mechanisms driving these gender-specific differences in recurrent stroke risk, further research is warranted. Exploring the interactions between age, gender, and other risk factors could offer valuable insights for tailoring effective stroke prevention strategies to specific populations.

Marital status significantly influenced recurrent stroke risk among working-age adults, particularly early-career workers. Married or widowed/divorced women in this age group faced a higher risk of recurrent stroke compared to single women, whereas no significant association was observed in mid-to-late-career workers. Previous studies indicate that marital transitions or changes in marital status can substantially affect stress levels and health behaviors, contributing to elevated stroke risk. ^34– 38^ This age-specific disparity could potentially be associated with increased stress, lifestyle changes, or other factors related to marital responsibilities among younger individuals. Further research is needed to better understand the mechanisms underlying these age-specific associations.

Fasting blood sugar (FBS) showed a significant association with an increased risk of recurrent stroke among early-career workers. Previous studies have indicated that individuals without a history of diabetes mellitus but with fasting blood sugar level >7mmol/L, or those with prediabetes, are considered at risk for developing recurrent stroke. ^39– 42^ However, in our study, no significant association between FBS and recurrent stroke was observed in mid-to-late-career workers. This finding suggests that FBS levels may not play a prominent role as a risk factor for recurrent stroke in the older age group, where other factors may have a more significant impact on stroke risk. Understanding these age-specific differences in this association is crucial for stroke management and prevention strategies. For early-career workers, early detection and management of elevated FBS levels may be critical in mitigating the risk of recurrent stroke.

Our findings revealed that current drinkers, both among younger and mid-to-late-career workers, had a higher risk of recurrent stroke compared to non-drinkers. While previous research has connected alcohol consumption to stroke recurrence in elderly patients, ^8^ its association with recurrent stroke in working-age adults is less clear. However, studies have suggested that heavy alcohol consumption is a modifiable risk factor for stroke in this age group. ^43– 46^ Addressing alcohol consumption is crucial in stroke prevention strategies for working-age adults. Healthcare providers should educate patients about the potential risks and promote healthier lifestyle choices. Understanding the specific patterns of alcohol consumption in different age groups can help tailor effective intervention programs. Reducing heavy alcohol intake and promoting responsible drinking behaviors may significantly lower the burden of recurrent stroke among working-age adults.

Our study found a significant association between sedentary lifestyle and increased recurrent stroke risk among early-career workers, whereas no significant association was observed in mid-to-late-career workers. Previous research supports that younger urban workers often spend more time engaged in sedentary activities due to work demands, digital leisure, and lifestyle preferences, potentially increasing their stroke risk. ^47– 50^ Interestingly, Vilhelmson et al. ^51^ further indicated that younger workers allocate significantly more time to work-related activities and digital leisure compared to older adults, who are more likely to engage in outdoor activities and regular exercise. Understanding these age-specific associations is essential for designing targeted stroke prevention strategies. For early-career workers, interventions aimed at reducing sedentary behaviors and promoting regular physical activity could effectively mitigate the risk of recurrent stroke. Implementing workplace wellness programs and encouraging regular breaks during the workday are recommended strategies for reducing sedentary behavior and its associated risks.

Our study identified a significant association between lack of workplace support for health and an increased risk of recurrent stroke among early-career workers, whereas no similar association was observed among mid-to-life-career workers. Previous studies have indicated that younger employees often encounter greater work-related pressures, including long hours, intense competition, and insufficient organizational support, contributing indirectly to unhealthy lifestyle behaviors and increased stroke risk. ^52– 54^ For instance, stressful workplace environments have been linked to poor dietary habits, decreased physical activity, increased alcohol intake, insufficient sleep, and chronic stress—factors closely tied to elevated stroke risk. ^55, 56^ Furthermore, younger employees, who frequently prioritize career advancement and productivity, may neglect health warnings and self-care practices, exacerbating their vulnerability. In contrast, mid-to-life-career workers typically exhibit more stable psychological well-being, better manage work-related stress, and adhere more consistently to health-promoting behaviors, thus mitigating these risks. ^57^ Understanding this age-specific difference is vital for designing targeted interventions. Workplace wellness initiatives and stress management programs tailored specifically to younger employees could effectively promote healthier lifestyles, reducing the likelihood of recurrent stroke in this demographic.

Our study revealed a significant association between workplace interpersonal relationships and recurrent stroke among working-age adults. Interestingly, we found that these relationships acted as a significant protective factor against recurrent stroke among early-career workers but did not seem to have the same impact on stroke risk for mid-to-late-career workers. Positive interpersonal relationships in the workplace may play a crucial role in buffering against the risk of recurrent stroke among early-career workers, contributing to reduced stress levels, improved mental well-being, and overall job satisfaction. ^58, 59^ As early-career workers often face the challenges of establishing themselves in their careers and navigating work-related stress, a positive social environment at work can act as a protective factor, influencing their health outcomes, including stroke risk. ^60^ In contrast, mid-to-late-career workers may have developed better coping mechanisms and emotional resilience, which could mitigate the impact of workplace relationships on their adverse health outcomes and promote well-being. ^61^ Understanding the differential influence of workplace interpersonal relationships on stroke risk in different age group is vital for designing targeted interventions. Reducing work-related stress could be a valuable component of stroke prevention strategies, particularly for early-career workers.

Examining these findings through the socio-ecological model ^62^ provides deeper insight into the intricate factors influencing urban areas. Individual behaviors like drinking habits and physical activity levels intersect with the broader urban landscape, including workplace dynamics and healthcare services accessibility. These interactions highlight the necessity for comprehensive, multi-level interventions that address both individual choices and the urban milieu. Effort to mitigate recurrent stroke risk among urban working-age adults should not solely concentrate on individual risk factors but should also consider the urban environment holistically. This involves promoting healthier behaviors, enhancing workplace health support, and improving healthcare services accessibility within the city. By aligning interventions with socio-ecological model principles, healthcare providers and policymakers can collaboratively craft a healthier urban environment, consequently alleviating the burden of recurrent stroke on this vulnerable population.

Several limitations of this study may influence the interpretation of the results. First, this study’s single-site hospital setting may limit the generalizability of the findings. While the study provides valuable insights into the specific urban setting, caution should be exercised when extrapolating these results to different geographic locations or diverse healthcare settings. To enhance external validity, future research should include larger and more diverse samples from multiple healthcare centers or regions. Second, a notable limitation lies in the absence of detailed information on medication use, physical activity, work-related stress, and the urban environment relevant to working-age adults. These factors can play crucial roles in influencing stroke risk and recurrence among this population. Collecting comprehensive data on these variables in future research would lead to a more profound understanding of their contributions to recurrent stroke risk, thereby guiding better-informed stroke prevention strategies. Lastly, it is essential to recognize that the risk factors examined in this study may interact with one another, leading to mutual influences and complex interplay. Comorbidities and the interactions between risk factors can significantly affect stroke recurrence risk, warranting thorough investigation. Future studies should aim to explore the synergistic effects of multiple risk factors and consider potential confounding variables to elucidate a more comprehensive picture of stroke recurrence risk among working-age adults.

## Conclusion

In conclusion, this study identified the key factors associated with recurrent stroke among working-age adults in urban Bangkok. Notably, high fasting blood sugar (aOR = 3.70, 95% CI: 1.66–8.27), alcohol consumption (aOR = 3.63, 95% CI: 2.01–6.54), sedentary lifestyle (aOR = 2.77, 95% CI: 1.50–5.13), and lack of workplace health support (aOR = 2.02, 95% CI: 1.13–3.63) were significantly associated with increased recurrence risk. These findings highlight the importance of targeting modifiable behavioral and occupational risk factors through tailored, age-specific prevention strategies.

Healthcare providers should prioritize evidence-based interventions such as lifestyle modification, alcohol reduction, increased physical activity, stress management, and regular health screenings. Multidisciplinary collaboration and workplace wellness initiatives may further enhance stroke prevention. Addressing these factors can reduce the risk of recurrence, minimize disability, and improve the quality of life of urban working-age populations.

## Declarations

### Ethical considerations

The study obtained approval from the Institutional Review Board (IRB) of the Faculty of Medicine Vajira Hospital, *Bangkok, Thailand (Approval* no. 110/64 E). The approval was granted on August 2, 2021, and is valid until August 1, 2022.

*Consent statement*

Before participation, all participants were informed about the study and their right to voluntary participation. They provided written informed consent prior to being enrolled in the study. All collected data was kept confidential and anonymous, ensuring the privacy of all participants. This study adhered to the principles outlined in the Declaration of Helsinki, ensuring ethical conduct.

## Author contribution statement

Yupha Wongrostrai contributed to conceptualization, data curation, formal analysis, funding acquisition, investigation, methodology, project administration, resources, software, supervision, validation, visualization, writing—original draft preparation, and writing—review and editing.

Araya Chiangkhong contributed to conceptualization, data curation, formal analysis, funding acquisition, investigation, methodology, project administration, resources, software, supervision, validation, visualization, writing—original draft preparation, and writing—review and editing.

Charin Suwanwong contributed to conceptualization, data curation, formal analysis, methodology, validation, visualization, writing—original draft preparation, and writing—review and editing.

Anon Khunakorncharatphong contributed to conceptualization, data curation, formal analysis, methodology, software, validation, visualization, writing—original draft preparation, and writing—review and editing.

## Additional information

No additional information is available for this paper.

## Data Availability

*Ethical and security consideration* The data consists of personal medical records of patients, and access is restricted to protect patient confidentiality. To apply for access to the data, readers or reviewers must submit a formal request including the purpose of the data use, a detailed research plan, and proof of ethical approval from a recognized institutional review board (IRB). Access will be granted only under the condition that the data will be used solely for the approved research purposes, and all necessary measures to ensure data privacy and security are in place. Applications should be directed to the corresponding author, and each request will be reviewed on a case-by-case basis.
