# Lifestyle and Occupational Factors Associated with Recurrent Stroke among Working-Age Adults in Urban Areas of Thailand

**DOI:** 10.12688/f1000research.154968.3

**Published:** 2025-06-05

**Authors:** Yupha Wongrostrai, Araya Chiangkhong, Charin Suwanwong, Anon Khunakorncharatphong

**Affiliations:** 1 Kuakarun Faculty of Nursing, Navamindradhiraj university, Bangkok, Thailand; 2 Behavioral Science Research Institute, Srinakharinwirot University, Bangkok, Thailand; 3 Faculty of Medicine Siriraj Hospital, Mahidol University, Bangkok, Thailand

**Keywords:** Recurrent stroke, Working-age, Adult, Urban, Thailand

## Abstract

**Background:**

Stroke survivors, particularly those of working age, are at an increased risk of recurrent stroke within one–five years of the initial event, largely due to suboptimal management of risk factors. This study aimed to identify lifestyle and occupational factors associated with recurrent stroke in this demographic population.

**Methods:**

This case-control study included 100 patients with recurrent ischemic stroke and 200 ischemic stroke survivors without recurrence, who were recruited from the hospital database. Multivariate logistic regression was used to identify significant factors associated with recurrence, which were presented as adjusted odds ratios (aORs) with 95% confidence intervals (CIs).

**Results:**

The mean age was 45.4 years (SD = 15.1) among cases and 50.6 years (SD = 6.5) among controls. The male-to-female ratios were 1.17:1 and 1.94:1 in the case and control groups, respectively. Significant factors associated with recurrent stroke included female sex (aOR: 1.83; 95% CI [1.10–3.29]), high fasting blood sugar (aOR: 3.70; 95% CI [1.66–8.27]), current alcohol consumption (aOR: 3.63; 95% CI [2.01–6.54]), sedentary lifestyle (aOR: 2.77; 95% CI [1.50–5.13]), and lack of workplace support for health (aOR: 2.02; 95% CI [1.13–3.63]). The associations between these factors and stroke recurrence varied according to the age group.

**Conclusions:**

This study highlights the critical role of modifiable lifestyle and occupational factors in stroke recurrence among working-age adults. Tailored age-specific prevention strategies—emphasizing physical activity, reduced alcohol use, and improved workplace health environments—may reduce the risk of recurrence and enhance health outcomes in this population.

## Introduction

Stroke remains a major global public health concern, ranking as the second leading cause of death and a substantial contributor to long-term disability. ^1^ Urban centers, such as Bangkok, have experienced a marked increase in stroke incidence, driven by shifts in socioeconomic conditions and urban environmental stressors. ^2, 3^ Thailand’s urban areas have undergone significant lifestyle transformations as the population transitions from agrarian to urban-centric living. ^4, 5^ Bangkok, in particular, exemplifies the dual challenges of rapid urbanization—offering improved access to healthcare while also introducing new health risks such as air pollution, urban heat-island effects, and psychosocial stress. ^6^

Recent national data report a stroke recurrence rate of 53.6% within one year, indicating a high burden of secondary stroke events in Thailand. ^7^ Recurrent strokes are often associated with poorly controlled modifiable risk factors, including excessive alcohol consumption, inadequate diabetes management, and uncontrolled hypertension. These factors contribute to increased mortality, frequent hospital readmissions, and long-term disability. ^8– 11^ While advanced age is a well-established predictor of recurrence, emerging evidence reveals a growing burden among working-age adults (18–60 years) in urban areas such as Bangkok. ^12– 14^

Although returning to work after stroke has been shown to improve well-being and social reintegration, ^15, 16^ urban occupational environments may paradoxically increase recurrence risk due to chronic stress, limited physical activity, and unhealthy lifestyle behaviors. Liangruenrom et al. identified strong associations between sedentary behavior and urban living in working-age Thai adults, highlighting the psychosocial and environmental challenges embedded in modern occupational contexts. ^17^

Furthermore, ischemic stroke accounts for approximately 80–85% of all stroke cases in Thailand, including among working-age adults in Bangkok. ^18^ Given this high prevalence and the lack of targeted evidence, the present study focuses specifically on recurrent ischemic stroke, with the primary outcome defined as recurrence within 12 months of the initial event.

Despite prior investigations into lifestyle and clinical risk factors for recurrent stroke in working-age adults, ^14, 19^ significant knowledge gaps remain—particularly regarding the influence of urban work-related stressors in this population. These include high job demands, limited workplace support, interpersonal conflicts, and sedentary working conditions. ^20, 21^ Understanding these factors is critical for developing effective, context-specific interventions to reduce stroke recurrence and improve the quality of life among this vulnerable demographic.

## Methods

### Setting and sample

This study employed a case-control design. Participants were classified based on their recurrent stroke status. The study included consecutive working-age adults (aged 20–60 years) who were diagnosed with **first-ever ischemic stroke** at the Faculty of Medicine Vajira Hospital, Bangkok, Thailand, between July 2020 and August 2021. Ischemic stroke was the most common stroke type observed in this setting, accounting for approximately 85% of all initial stroke cases during the study period.

**Cases** were defined as patients who had experienced a **recurrent ischemic stroke**, confirmed by a neurologist using computerized tomography (CT) or magnetic resonance imaging (MRI). Recurrent stroke was defined as the occurrence of a new focal neurological deficit lasting more than 24 hours and clinically distinct from the index stroke. To differentiate true recurrence from stroke progression, only events that occurred more than 28 days after the initial ischemic stroke were considered recurrent, based on definitions used in prior epidemiological studies. ^22^

**Controls** were ischemic stroke survivors without any documented recurrence who attended regular follow-up visits at the hospital’s neurology outpatient department during the same period. Controls were randomly selected from the hospital registry and matched according to the inclusion criteria.

Both cases and controls were required to have continuously resided in the urban areas of Bangkok for at least five years prior to participation. **Exclusion criteria** included unemployment at the time of recruitment, loss of consciousness at stroke onset, hemorrhagic stroke, hemorrhagic transformation, **transient ischemic attack (TIA)**, or other significant neurological impairments that could affect accurate behavioral recall.

Patients with TIA were excluded because TIA is characterized by transient neurological symptoms without lasting deficits or confirmatory imaging findings, which can complicate the classification of stroke recurrence.

Participants were stratified into age groups (25–40 and 41–60 years) based on occupational health literature, which classifies these age bands into early- and mid-to-late-career stages. ^23, 24^

The sample size was calculated using Epi Info software version 7.2.5.0 (CDC, Atlanta, USA), available at: https://www.cdc.gov/epiinfo/index.html.The calculation used a double population formula suitable for an unmatched case-control study, based on a recurrent stroke rate among controls of 50.5%, and an adjusted odds ratio (aOR) of 0.44, derived from a prior study conducted in India. ^25^ To achieve a 95% confidence interval (CI) with 80% statistical power and maintain a controls-to-cases ratio of 2:1. The initial sample size was 250. An additional 20% was added to account for potential non-responses, resulting in a final total of 300 participants (100 cases and 200 controls).

### Instruments

A structured questionnaire was developed based on a literature review of stroke prevention guidelines. ^26^ The tool consists of items covering three main domains: demographic characteristics (age, gender, and marital status), health-related behaviors, and occupational factors. Health-related behaviors were retrospectively assessed over the past year and categorized into four domains: (i) preventive health behavior, (ii) smoking status, (iii) drinking status, and (iv) a sedentary lifestyle.

The questionnaire covered demographic characteristics (age, gender, and marital status), health-related behaviors (preventive health behavior, smoking status, drinking status, and sedentary lifestyle), and occupational factors (interpersonal relationships at the workplace, job characteristics, and physical work environment). Clinical characteristics, such as stroke subtypes, fasting blood sugar (FBS), body mass index (BMI), hypertension, diabetes mellitus, and dyslipidemia, were obtained from medical records. Health-related behaviors, reflecting the past year, were assessed following key guidelines and insights from the literature. Health-related behaviors, gathered retrospectively over the past year, were assessed following the key recommendations and insights from the literature and were organized into four domains: (i) preventive health behavior, (ii) smoking status, (iii) drinking status, and (iv) sedentary lifestyle. Preventive health behavior was measured by compliance with recommended preventive measures, such as medication adherence, physical activity, regular physical examinations, sufficient sleep, maintaining a healthy weight, and a healthy diet (13 items). Participants rated their responses on a 3-point Likert scale ranging from ‘always’ to ‘never’. The Cronbach’s alpha for preventive health behavior was 0.79. Smoking status was determined by asking participants if they currently smoked. The response categories were ‘never’, ‘ever’, and ‘yes’. Participants were classified as non-smokers (never and ever combined) and current smokers. Drinking status was assessed by asking participants if they currently drank alcohol. The response categories were ‘never’, ‘ever’, and ‘yes’. Participants were classified as non-drinkers (never and ever combined) and current drinkers. The sedentary lifestyle was assessed using a 6-item scale specifically developed for this study, which was informed by the definitions of sedentary behavior outlined by Tremblay et al. ^27^ This scale includes activities such as lying down, reclining, and sitting. Participants responded to the items using a 4-point Likert scale ranging from ‘always’ to ‘never.’ The internal consistency of the sedentary lifestyle scale was measured, yielding a Cronbach’s alpha of 0.70.

The measurement tool for occupational characteristics was developed based on a thorough review of the relevant literature, which identified three primary domains for assessment: interpersonal relationships in the workplace, job characteristics, and the physical work environment. Interpersonal relationships were evaluated by examining the quality of interactions with colleagues and supervisors, ^28, 29^ employing five specific items that pertain to job autonomy, job feedback, task significance, task identity, and skill variety. Participants rated their experiences using a 3-point Likert scale, ranging from ‘always’ to ‘never.’ The physical work environment was defined according to participants’ perceptions of several factors, including lighting, noise, temperature, and workplace support for health, assessed through four items. Responses were categorized as ‘no’ or ‘yes.’ The Cronbach’s alpha for job characteristics was determined to be 0.79, indicating acceptable internal consistency. Clinical characteristics data were collected from medical records. Stroke subtypes were classified into categories such as embolic, thrombotic, lacunar, and uncertain. ^30^

Current fasting blood sugar (FBS) levels were measured using an oxidase enzymatic method. FBS was measured in mg/dL using the oxidase enzymatic method. FBS was categorized as: Normal (<100 mg/dL), Medium (100–125 mg/dL), and High (≥126 mg/dL), based on ADA guidelines. Body mass index (BMI) was calculated as weight in kilograms divided by height in meters squared. Additionally, historical FBS and BMI data from 6 to 12 months prior were collected for comparison. Hypertension, diabetes mellitus, and dyslipidemia were defined based on physician diagnoses.

### Data collection procedure

Data were collected through face-to-face interviews conducted by nurses specializing in stroke care, using the structured questionnaire described previously. These nurses underwent comprehensive training encompassing the study objectives, questionnaire content, ethical considerations, and standardized procedures for data collection. All the completed questionnaires were reviewed daily by senior investigators to ensure data accuracy, completeness, and consistency.

The participants provided information regarding their stroke history, date of diagnosis, clinical manifestations, lifestyle-related risk factors, and family history of chronic diseases. Physical examination was conducted to assess height, weight, and blood pressure. Clinical data—including stroke subtypes, FBS, BMI, and comorbid conditions—were extracted directly from medical records. Each participant was followed for a period of 12 months from the date of the initial ischemic stroke diagnosis. Recurrent stroke events were identified through clinical documentation and follow-up visits within this period.

### Data analysis

Data were analyzed using STATA 17 software (Serial number: 501706420821).for univariate, bivariate, and multivariate analyses. The chi-square test compared the distribution of all variables in the univariate analysis. Univariate odd ratios (ORs) were calculated for factors with a significant difference ( *p-value* < 0.2). ^29^ Factors meeting this criterion were included in the multivariate analysis, which used logistic regression to calculate adjusted odds ratios (aORs) and 95% confidence interval (CIs) to identify significant factors associated with recurrent stroke. A *p*- *value* < 0.05 indicated statistical significance.

## Results

**Figure 1.**
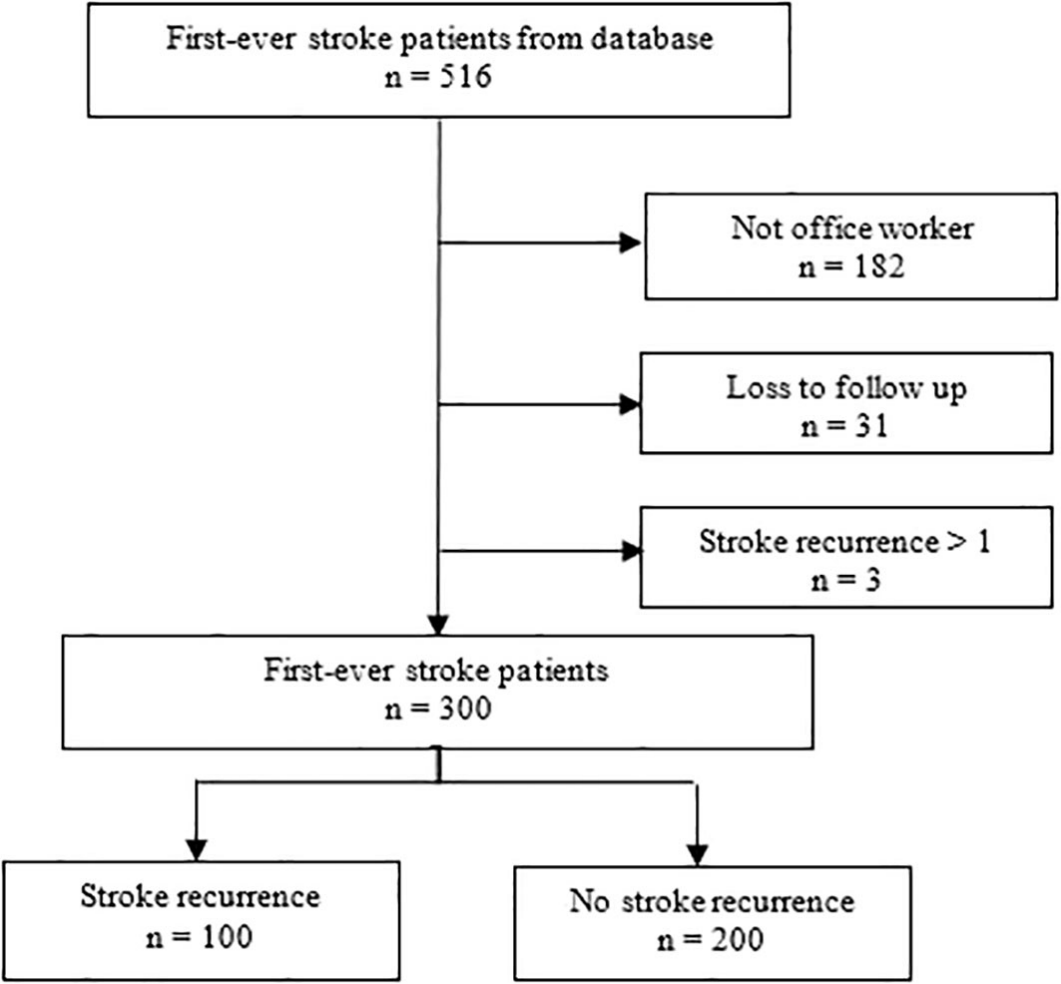
Flowchart of participant selection process (patients with more than one stroke recurrence were excluded).

From a total of 516 eligible first-ever stroke patients initially identified from the hospital database, 300 patients who met all inclusion criteria were included in this study. The mean age was 45.4 years (SD = 15.1) in the case group and 50.6 years (SD = 6.5) in the control group. The male-to-female ratio was 1.17:1 among cases and 1.94:1 among controls. Figure 1 illustrates the detailed recruitment and participant selection processes, showing reasons for exclusion such as non-office workers (n=182), loss to follow-up (n=31), and patients with more than one stroke recurrence (n=3).

A total of 300 participants were included in the study: 100 patients with recurrent stroke and 200 controls without recurrence. The mean age of 43.1 years in the case group and 44.2 years in the control group. In terms of sex distribution, 54.0% of the cases and 66.0% of the controls were male, corresponding to male-to-female ratios of 1.17:1 and 1.94:1, respectively. Most participants were married (64.0% in cases and 57.0% in controls). Statistically significant differences between the two groups were found in sex, marital status, fasting blood sugar (FBS), body mass index (BMI), hypertension, diabetes mellitus, dyslipidemia, smoking, alcohol consumption, sedentary lifestyle, interpersonal relationships at the workplace, and workplace support for health. The baseline characteristics of the participants are summarized in Table 1.

**Table 1.** Baseline characteristics of the study participants (n=300).

Characteristics	Case (n = 100)	Control (n = 200)	*p-value *
	n (%)	n (%)	
Age			.28
25-40 years	66 (66.0)	119 (59.5)	
41-60 years	34 (34.0)	81 (40.5)	
*M*+ *SD*	45.4 ± 15.1	50.6 ± 6.5	
Gender			<.05
Male	54 (54.0)	132 (66.0)	
Female	46 (46.0)	68 (34.0)	
Male-to-Female Ratio	1.94:1	1.17:1	
Marital status			<.01
Single	18 (18.0)	70 (35.0)	
Married	64 (64.0)	114 (57.0)	
Widowed/Divorced	18 (18.0)	16 (8.0)	
Fasting Blood Sugar (FBS)			<.01
Normal	21 (21.4)	59 (29.5)	
Medium	34 (34.7)	100 (50.0)	
Hight	43 (43.9)	41 (20.5)	
Body Mass Index (BMI)			<.01
Normal weight	33 (33.0)	107 (53.5)	
Overweight	67 (67.0)	93 (46.5)	
Hypertension			<.05
Yes	67 (67.0)	109 (54.5)	
No	33 (33.0)	91 (45.5)	
Diabetes mellitus			<.05
Yes	50 (50.0)	74 (37.0)	
No	50 (50.0)	126 (63.0)	
Dyslipidemia			<.05
Yes	61 (61.0)	94 (47.0)	
No	39 (39.0)	106 (53.0)	
Smoking status			<.05
Current smoker	47 (47.0)	69 (34.5)	
Non-smoker	53 (53.0)	131 (65.5)	
Drinking status			<.01
Current drinker	55 (55.0)	54 (27.0)	
Non-drinker	45 (45.0)	146 (73.0)	
Preventive health behavior			.51
Low	42 (42.0)	92 (46.0)	
High	58 (58.0)	108 (54.0)	
Sedentary lifestyle			<.01
Low	46 (46.0)	152 (76.0)	
High	54 (54.0)	48 (24.0)	
Interpersonal relationship at workplace			<.01
Low	51 (51.0)	67 (33.5)	
High	49 (49.0)	133 (66.5)	
Job characteristics			.17
Low	42 (42.0)	99 (49.5)	
High	58 (58.0)	101 (50.5)	
Physical work environment			
Light			.57
Enough	50 (50.0)	93 (46.5)	
Poor/too much	50 (50.0)	107 (53.5)	
Noise			.86
Yes	33 (33.0)	68 (34.0)	
No	67 (67.0)	132 (66.0)	
Heat and cold stress			.46
Yes	24 (24.0)	56 (28.0)	
No	76 (76.0)	144 (72.0)	
Workplace support for health			<.01
Yes	54 (54.0)	74 (37.0)	
No	46 (46.0)	126 (63.0)	

^a^ chi-squared for proportions.

Based on the significant differences in the baseline characteristics, specific variables were selected for inclusion in the multivariate analysis model. The results, presented in Table 2, showed that female gender (aOR = 1.83, 95% CI: 1.01, 3.29), high FBS (aOR = 3.70, 95% CI: 1.66, 8.27), drinking status (aOR = 3.63, 95% CI: 2.01, 6.54), sedentary lifestyle (aOR = 2.77, 95% CI: 1.50, 5.13), and lack of workplace support for health (aOR = 2.02, 95% CI: 1.13, 3.63) were significantly associated with recurrent stroke among the working-age adults.

**Table 2.** Logistic regression of predictors for recurrent stroke (n=300).

Predictors	Crude OR (95% CI)	*p*-value	Adjusted OR (95% CI) *	*p*-value
Gender (Female vs. Male *)	1.65 (1.01, 2.70)	<.05	1.83 (1.01, 3.29)	<.05
Marital status (vs. Single *)				.09
Married	2.18 (1.20, 3.98)	<.05	1.65 (0.81, 3.36)	.17
Widowed/Divorced	4.38 (1.87, 10.23)	<.01	3.13 (1.09, 8.94)	<.05
Fasting Blood Sugar (FBS) (vs. Normal *)				<.01
Medium	0.96 (0.51, 1.80)	.89	1.05 (0.50, 2.18)	.90
High	3.08 (1.60, 5.93)	<.01	3.70 (1.66, 8.27)	<.01
Body Mass Index (BMI) (Normal vs. Overweight *)	2.34 (1.42, 3.85)	<.01	1.76 (0.96, 3.21)	.07
Hypertension (Yes vs. No *)	1.70 (1.03, 2.80)	<.05	1.83 (0.99, 3.37)	.05
Diabetes mellitus (Yes vs. No *)	1.70 (1.05, 2.77)	<.05	1.38 (0.76, 2.50)	.29
Dyslipidemia (Yes vs. No *)	1.76 (1.08, 2.87)	<.05	0.80 (0.43, 1.49)	.49
Smoking status (Current vs. Non-smoker *)	1.68 (1.03, 2.75)	<.01	1.57 (0.87, 2.85)	.14
Drinking status (Current vs. Non-drinker *)	3.30 (2.00, 5.46)	<.01	3.63 (2.01, 6.54)	<.01
Sedentary Lifestyle (High vs. Low *)	2.70 (1.62, 4.49)	<.01	2.77 (1.50, 5.13)	<.01
Interpersonal relationship at workplace (High vs. Low *)	0.48 (0.30, 0.79)	<.01	0.56 (0.31, 1.01)	.05
Workplace support for health (No vs. Yes *)	2.00 (1.23, 3.25)	<.01	2.02 (1.13, 3.63)	<.05

* Adjusted for gender, marital status, FBS, BMI, hypertension, diabetes mellitus, dyslipidemia, smoking, drinking, sedentary lifestyle, interpersonal relationships at the workplace, and workplace support for health.

Various factors contributed to recurrent stroke were analyzed into two age groups: individuals aged 25-40 years (early-career workers) and those aged 41-60 years (mid-to-late-career workers) ( Table 3). Among early-career workers, the most significant factors influencing recurrent stroke were being widowed/divorced (aOR = 7.62, 95% CI: 1.87, 31.12), drinking status (aOR = 4.28, 95% CI: 1.87, 9.80), sedentary lifestyle (aOR = 4.27, 95% CI: 1.84, 9.88), high FBS (aOR = 4.10, 95% CI: 1.32, 12.77), female gender (aOR = 3.28, 95% CI: 1.49, 7.25), being married (aOR = 2.59, 95% CI: 1.02, 6.55), and lack of workplace support for health (aOR = 2.35, 95% CI: 1.05, 5.23). Interestingly, having high interpersonal relationships at the workplace appeared to have a protective effect against recurrent stroke (aOR = 0.34, 95% CI: 0.15, 0.76). On the other hand, among mid-to-late-career workers, only drinking status was found to be associated with recurrent stroke (aOR = 3.38, 95% CI: 1.17, 9.72).

**Table 3.** Logistic regression of predictors for recurrent stroke between aged 25-40 (n=66) years and 41-60 years (n=34).

Predictors	Cases n (%)	Aged 25-40 year (n = 66)				Cases n (%)	Aged 41-60 year (n = 34)			
		Crude OR (95% CI)	* p-value *	Adjusted OR (95% CI)	*p-value *		Crude OR (95% CI)	* p-value *	Adjusted OR (95% CI)	* p-value *
Gender (Female vs. Male)	28 (42.42)	2.89 (1.55, 5.39)	<.01	3.28 (1.49, 7.25)	<.01	18 (52.94)	0.52 (0.21, 1.30)	.16	0.45 (0.14, 1.50)	.19
Marital status (vs. Single *)					<.01					.83
Married	40 (60.61)	3.44 (1.60, 7.40)	<.01	2.59 (1.02, 6.55)	<.05	24 (70.59)	0.96 (0.35, 2.64)	.94	0.71 (0.20, 2.56)	.60
Widowed/Divorced	10 (15.20)	9.20 (3.14, 26.98)	<.01	7.62 (1.87, 31.12)	<.01	5 (14.71)	0.86 (0.17, 4.23)	.85	0.55 (0.06, 5.00)	.60
Fasting Blood Sugar (FBS) (vs. Normal *)					<.05				<.05
Medium	20 (30.30)	0.88 (0.41, 1.88)	.74	1.60 (0.62, 4.17)	.33	15 (44.12)	0.99 (0.31, 3.20)	.99	0.76 (0.17, 3.41)	.72
High	20 (30.30)	2.62 (1.13, 6.08)	<.05	4.10 (1.32, 12.77)	<.05	15 (44.12)	4.33 (1.47, 12.79)	<.01	4.07 (0.83, 19.98)	.08
Body Mass Index (BMI) (Normal vs. Overweight *)	35 (53.03)	2.25 (1.21, 4.19)	<.05	1.95 (0.85, 4.44)	.11	13 (38.24)	2.71 (1.13, 6.52)	<.05	1.53 (0.51, 4.58)	.45
Hypertension (Yes vs. No *)	30 (45.45)	1.47 (0.80, 2.70)	.22	1.87 (0.84, 4.15)	.12	17 (50.00)	2.89 (1.08, 7.78)	<.05	2.44 (0.68, 8.79)	.16
Diabetes mellitus (Yes vs. No *)	28 (42.42)	1.61 (0.88, 2.96)	.12	1.25 (0.56, 2.81)	.59	17 (50.00)	1.77 (0.78, 4.04)	.18	2.08 (0.64, 6.77)	.22
Dyslipidemia (Yes vs. No *)	25 (37.88)	1.44 (0.78, 2.63)	.24	0.53 (0.23, 1.23)	.13	18 (52.94)	2.72 (1.15, 6.40)	<.05	1.08 (0.28, 4.18)	.91
Smoking status (Current vs. Non-smoker *)	27 (40.91)	1.21 (0.66, 2.24)	.54	1.15 (0.50, 2.60)	.75	16 (47.06)	3.01 (1.31, 6.89)	<.01	2.75 (0.99, 7.62)	.05
Drinking status (Current vs. Non-drinker *)	36 (54.55)	2.83 (1.52, 5.27)	<.01	4.28 (1.87, 9.80)	<.01	19 (55.88)	4.24 (1.79, 10.01)	<.01	3.38 (1.17, 9.72)	<.05
Sedentary Lifestyle (High vs. Low *)	35 (53.03)	3.41 (1.81, 6.42)	<.01	4.27 (1.84, 9.88)	<.01	13 (38.24)	1.57 (0.63, 3.90)	.33	1.57 (0.43, 5.80)	.50
Interpersonal relationship at workplace (High vs. Low *)	25 (37.88)	0.45 (0.24, 0.84)	<.05	0.34 (0.15, 0.76)	<.01	21 (61.76)	0.48 (0.21, 1.09)	.08	0.96 (0.33, 2.80)	.94
Workplace support for health (No vs. Yes *)	29 (43.94)	2.32 (1.25, 4.29)	<.01	2.35 (1.05, 5.23)	<.05	16 (47.06)	1.66 (0.74, 3.73)	.22	2.47 (0.81, 7.53)	.11

* Adjusted for gender, marital status, FBS, BMI, hypertension, diabetes mellitus, dyslipidemia, smoking, drinking, sedentary lifestyle, interpersonal relationships at the workplace, and workplace support for health.

## Discussion

Working-age adults in Bangkok, Thailand, who experience a first-ever stroke face a heightened risk of recurrence, largely driven by lifestyle and occupational factors. This investigation sought to identify key determinants of recurrent stroke within this demographic. Findings indicated that female gender, elevated fasting blood sugar (FBS), alcohol use, physical inactivity, and insufficient workplace health support were significant contributors. Notably, the influence of these factors varied between early-career and mid-to-late-career individuals.

Among these, female gender emerged as a significant predictor of recurrent stroke among working-age adults, with a notably stronger association observed in early-career individuals. In contrast, no meaningful link was found among those in mid-to-late career stages. Previous research suggests that younger urban women may be particularly susceptible to certain risk factors, including unhealthy lifestyle behaviors, environmental exposures, and occupational stress. ^31– 35^ These disparities likely stem from such influences. Further investigation into the interplay of gender and contributing variables is essential to deepen understanding and inform the development of targeted prevention strategies.

Building on the role of sociodemographic factors, marital status was a notable factor influencing stroke recurrence among working-age adults, particularly those in the early stages of their careers. Women who were married, widowed, or divorced faced a higher risk compared to their single counterparts; a pattern not observed among mid-to-late-career individuals. Prior research suggests that marital transitions can significantly impact stress levels and health-related behaviors, potentially increasing vulnerability to stroke. ^36– 40^ This finding underscores the importance of considering psychosocial stressors and their relationship to stroke risk among working-age populations.

Adding to the physiological risk profile, elevated fasting blood sugar (FBS) was significantly linked to recurrent stroke among early-career workers. Prior research has shown that individuals with prediabetes or FBS levels exceeding 7 mmol/L, even without a diabetes diagnosis, face increased risk of stroke recurrence. ^41– 44^ In contrast, this association was not evident among mid-to-late-career individuals, suggesting that glycemic status may be a less influential factor in older age groups, where other variables likely play a larger role. These results highlight the role of metabolic health in stroke prevention and emphasize the need for early screening and intervention to manage glycemic levels in this demographic.

Behavioral risk factors further illustrated the complexity of recurrence patterns. Current alcohol consumption was associated with an elevated risk of recurrent stroke across both early- and mid-to-late-career workers. Although prior studies have established this link in older adults, 8 its relevance to the working-age population remains less well defined. Emerging evidence, however, supports heavy drinking as a modifiable risk factor in this group. ^45– 48^ Incorporating alcohol-related risk reduction into prevention strategies is essential. Healthcare professionals should raise awareness about the dangers of excessive intake and encourage healthier behavioral choices.

Closely tied to behavioral habits, a sedentary lifestyle was significantly linked to recurrent stroke among early-career workers, while no such association was found in their older counterparts. Research suggests that younger urban employees often engage in prolonged inactivity due to occupational demands, screen-based leisure, and lifestyle habits. ^49– 52^ Vilhelmson et al. ^53^ further reported that this group dedicates more time to work-related tasks and digital entertainment, whereas older adults tend to participate more in outdoor and physical activities. Encouraging regular movement through wellness programs and scheduled activity breaks may help mitigate this risk.

Beyond individual behaviors, organizational factors also played a crucial role. Lack of workplace health support was significantly associated with recurrent stroke among early-career workers, a pattern not observed in mid-to-late-career individuals. Prior studies suggest that younger employees often face heightened job-related pressures—such as long hours, competitive environments, and limited organizational backing—which can indirectly lead to unhealthy behaviors and elevated stroke risk. ^54– 56^ Stressful work settings have been linked to poor diet, reduced physical activity, excessive alcohol use, sleep deprivation, and chronic stress. ^57, 58^ Additionally, the drive for career advancement may cause younger workers to overlook early health warnings and neglect self-care. In contrast, older employees tend to manage stress more effectively and engage in consistent health-promoting behaviors, reducing their vulnerability. ^59^ Wellness programs and stress management initiatives tailored to the needs of younger staff may foster healthier routines and help prevent stroke recurrence.

Complementing the organizational context, workplace interpersonal relationships were significantly associated with recurrent stroke risk among working-age adults, serving as a protective factor particularly for those in the early stages of their careers. This association was not evident among mid-to-late-career workers. Supportive social connections in the workplace may help buffer stress, enhance mental well-being, and improve job satisfaction, thereby reducing health risks such as stroke. ^60, 61^ Given that early-career individuals often face the pressures of establishing themselves professionally, a positive work environment can play a critical role in mitigating stress-related health outcomes. ^62^ In contrast, more experienced workers may rely on established coping strategies and emotional resilience, diminishing the impact of social dynamics on their well-being. ^63^ Designing interventions that foster inclusive, low-stress, and collaborative workplace cultures may support long-term prevention goals.

It is also worth noting that several commonly recognized risk factors—such as BMI, hypertension, diabetes mellitus, dyslipidemia, smoking status, and interpersonal relationships in the workplace—did not show significant associations with stroke recurrence in this study. While this may reflect true non-association within this specific population, it is also possible that subgroup distributions, limited sample sizes, or low variability in exposure contributed to the lack of significance. These findings should be interpreted with caution, and further studies with larger and more balanced samples are warranted to explore these factors more comprehensively.

Synthesizing these findings through the lens of the socio-ecological model ^64^ provides a deeper understanding of the complex interplay between personal behaviors and environmental influences in urban settings. Lifestyle factors such as alcohol use and physical inactivity intersect with broader elements like workplace culture and access to healthcare services. These multilayered interactions underscore the need for integrated interventions that address both individual and systemic determinants of health. Efforts to reduce recurrent stroke among urban working-age adults should move beyond personal risk factors to encompass the wider urban context. This includes fostering healthier routines, strengthening organizational support, and improving service accessibility. Aligning prevention strategies with socio-ecological principles enables healthcare professionals and policymakers to collaboratively shape healthier urban environments and ease the burden of stroke recurrence in vulnerable groups.

Several limitations should be considered when interpreting the findings. First, the use of a single-site hospital sample may restrict the generalizability of the results. Although the study offers meaningful insights into an urban population, caution is warranted when applying these outcomes to other geographic areas or healthcare contexts. Future research should aim to include larger, more diverse samples drawn from multiple institutions or regions to enhance external validity. Second, the lack of detailed data on medication usage, physical activity, occupational stress, and environmental conditions limits a comprehensive assessment of contributing factors. These elements are likely to play significant roles in stroke risk and recurrence among working-age adults. Gathering more robust data on these variables in future investigations would deepen understanding and inform more effective prevention strategies. Lastly, the interplay among risk factors—such as comorbidities and behavioral patterns—was not fully explored. Investigating how these variables interact could provide a more nuanced view of recurrence risk and support the development of multifaceted interventions.

## Conclusion

In conclusion, this study identified the key factors associated with recurrent stroke among working-age adults in urban Bangkok. The findings point to high fasting blood sugar, alcohol consumption, sedentary lifestyle, and lack of workplace health support as significant contributors to recurrence risk. These results underscore the critical need to address modifiable behavioral and occupational risk factors through targeted, age-sensitive prevention strategies.

Healthcare providers should prioritize evidence-based interventions, including lifestyle modifications, alcohol reduction, increased physical activity, stress management, and routine health screenings. Promoting multidisciplinary collaboration and implementing workplace wellness initiatives can further strengthen stroke prevention efforts. By addressing these key risk factors, it is possible to reduce recurrence rates, minimize long-term disability, and enhance the overall quality of life for urban working-age populations.

## Declarations

### Ethical considerations

The study obtained approval from the Institutional Review Board (IRB) of the Faculty of Medicine Vajira Hospital, *Bangkok, Thailand (Approval* no. 110/64 E). The approval was granted on August 2, 2021, and is valid until August 1, 2022.

*Consent statement*

Before participation, all participants were informed about the study and their right to voluntary participation. They provided written informed consent prior to being enrolled in the study. All collected data was kept confidential and anonymous, ensuring the privacy of all participants. This study adhered to the principles outlined in the Declaration of Helsinki, ensuring ethical conduct.

## Author contribution statement

Yupha Wongrostrai contributed to conceptualization, data curation, formal analysis, funding acquisition, investigation, methodology, project administration, resources, software, supervision, validation, visualization, writing—original draft preparation, and writing—review and editing.

Araya Chiangkhong contributed to conceptualization, data curation, formal analysis, funding acquisition, investigation, methodology, project administration, resources, software, supervision, validation, visualization, writing—original draft preparation, and writing—review and editing.

Charin Suwanwong contributed to conceptualization, data curation, formal analysis, methodology, validation, visualization, writing—original draft preparation, and writing—review and editing.

Anon Khunakorncharatphong contributed to conceptualization, data curation, formal analysis, methodology, software, validation, visualization, writing—original draft preparation, and writing—review and editing.

## Additional information

No additional information is available for this paper.

## Data Availability

*Ethical and security consideration* The data consists of personal medical records of patients, and access is restricted to protect patient confidentiality. To apply for access to the data, readers or reviewers must submit a formal request including the purpose of the data use, a detailed research plan, and proof of ethical approval from a recognized institutional review board (IRB). Access will be granted only under the condition that the data will be used solely for the approved research purposes, and all necessary measures to ensure data privacy and security are in place. Applications should be directed to the corresponding author, and each request will be reviewed on a case-by-case basis.
