# Supplementary material for: Lifestyle and Occupational Factors Associated with Recurrent Stroke among Working-Age Adults in Urban Areas of Thailand
Source: F1000Res. 2025 Jun 5;13:1445. Originally published 2024 Nov 29. [Version 3] doi: 10.12688/f1000research.154968.3 (PMC12179587; doi:10.12688/f1000research.154968.3)
Supplement: Peer review history

# Reviewer report: Reviewer response for version 3

**DOI:** 10.5256/f1000research.183217.r390104

**Published:** 2025-06-19

**Authors:** Anthony Rudd

**Affiliations:** 1 King’s College, London, UK

Previous reviews have highlighted some of the concerns about the paper and these have already been addressed satisfactorily by the authors. The data presented are of interest, but the limitations of the methodology need to be discussed in more detail. 

 It is acknowledged that the sample is just from one hospital and is not population based and there are limited data on medication compliance, but these are very important issues that do detract from the overall message. The control group are selected from patients attending the hospital for follow-up. By definition therefore they will be people who are complying with at least some aspects of stroke prevention compared to patients who do not attend for follow-up. The lack of data on use of medication (especially anti-platelets, anticoagulants and statins) and the lack of data on whether risk factors (especially hypertension) have actually been controlled are also significant weaknesses that need stronger acknowledgement. These are the factors that previous studies on stroke recurrence have shown to be of particular importance.

Is the work clearly and accurately presented and does it cite the current literature?

Yes

If applicable, is the statistical analysis and its interpretation appropriate?

Yes

Are all the source data underlying the results available to ensure full reproducibility?

Yes

Is the study design appropriate and is the work technically sound?

Yes

Are the conclusions drawn adequately supported by the results?

Partly

Are sufficient details of methods and analysis provided to allow replication by others?

Yes

Reviewer Expertise:

Stroke management and epidemiology

I confirm that I have read this submission and believe that I have an appropriate level of expertise to confirm that it is of an acceptable scientific standard, however I have significant reservations, as outlined above.

# Reviewer report: Reviewer response for version 3

**DOI:** 10.5256/f1000research.183217.r389967

**Published:** 2025-06-13

**Authors:** Lamia M’barek

**Affiliations:** 1 Department of Neurology, China National Clinical Research Center for Neurological Diseases, Beijing Tiantan Hospital, Capital Medical University, Beijing, China

I have reviewed the revisions made to the manuscript and I fully accept the changes. I have no further comments.

 Thank you for your hard work on this manuscript.

Is the work clearly and accurately presented and does it cite the current literature?

Yes

If applicable, is the statistical analysis and its interpretation appropriate?

Yes

Are all the source data underlying the results available to ensure full reproducibility?

Partly

Is the study design appropriate and is the work technically sound?

Partly

Are the conclusions drawn adequately supported by the results?

Yes

Are sufficient details of methods and analysis provided to allow replication by others?

Partly

Reviewer Expertise:

Clinical Research for Neurological Diseases

I confirm that I have read this submission and believe that I have an appropriate level of expertise to confirm that it is of an acceptable scientific standard.

# Reviewer report: Reviewer response for version 2

**DOI:** 10.5256/f1000research.179234.r386824

**Published:** 2025-05-26

**Authors:** Lamia M’barek

**Affiliations:** 1 Department of Neurology, China National Clinical Research Center for Neurological Diseases, Beijing Tiantan Hospital, Capital Medical University, Beijing, China

**Revision 2:**

 We would like to thank the authors for their initial revisions and the improvements made to enhance the manuscript quality. The following suggestions are provided to further strengthen the study:

**1. Clarify the Type of Stroke Included in the Study**

**Suggestion:** Explicitly state whether the study focuses on ischemic stroke, hemorrhagic stroke, or other types of stroke. If multiple types are included, provide a breakdown of the distribution of stroke types in the study population.

**2. Strengthen the Introduction**

**Primary Stroke Type Onset:** Define the most common type of stroke onset in the study population and provide its exact frequency (e.g., "Ischemic stroke accounted for 70% of initial strokes in our cohort").

**Recurrence Definition:** Clearly specify the type(s) of recurrent stroke used as the primary outcome (e.g., "The primary outcome was recurrent ischemic stroke within 12 months of the index event").

**3. Refine the Methodology Section**

**Definition of Recurrent Stroke:** If the study includes ischemic stroke, ensure the definition of recurrent stroke aligns with established criteria. If other stroke types are included, add appropriate definitions for each.

**4. Explain the Exclusion of TIA Patients**

**Rationale for Exclusion:** Provide a clear explanation for excluding patients with transient ischemic attack (TIA).

**5. Specify Follow-Up Period in Data Collection**

**Follow-Up Timeline:** Mention the duration of follow-up.

**6. Enhance Table 3 Presentation**

**Add Details:** Include the number and percentage of cases in each variable according to group subtype. As well, specify which factors were adjusted for in multivariate analyses.

**Improve the Discussion Section**

**Focus on Main Aim:** Avoid limiting the discussion to age-related risk factors unless it is central to the study’s objectives. Instead, focus on factors directly linked to the primary aim.

**Address Non-Significant Findings:** Acknowledge that non-significant results may relate to patient distribution.

**Revise Repetitive Language**

**Avoid "Our Study"**. Ensure paragraphs flow logically by connecting ideas.

**Simplify the Conclusion**

 Avoid citing odds ratios (OR) in the conclusion. Instead, highlight the major risk factors identified.

Is the work clearly and accurately presented and does it cite the current literature?

Yes

If applicable, is the statistical analysis and its interpretation appropriate?

Yes

Are all the source data underlying the results available to ensure full reproducibility?

Partly

Is the study design appropriate and is the work technically sound?

Partly

Are the conclusions drawn adequately supported by the results?

Yes

Are sufficient details of methods and analysis provided to allow replication by others?

Partly

Reviewer Expertise:

Clinical Research for Neurological Diseases

I confirm that I have read this submission and believe that I have an appropriate level of expertise to confirm that it is of an acceptable scientific standard, however I have significant reservations, as outlined above.

# Reviewer report: Reviewer response for version 1

**DOI:** 10.5256/f1000research.170076.r364042

**Published:** 2025-02-26

**Authors:** Norsima Nazifah Sidek

**Affiliations:** 1 Department of Community Medicine, School of Medical Sciences, Universiti Sains Malaysia, Clinical Research Centre, Hospital Sultanah Nur Zahirah, Ministry of Health Malaysia, Kelantan, Terengganu, Malaysia

- Some findings in the **discussion section** could be more critically analysed, linking to existing literature more explicitly.
- Ensure that references support all claims made in the manuscript. 
- More details on **how confounders were controlled** in the logistic regression analysis would strengthen the methodology section.
- Please explain more detail regarding the sampling method .
- There are several type of randomization. 
- Please explain more details  the validation process for the questionnaire 

Is the work clearly and accurately presented and does it cite the current literature?

Partly

If applicable, is the statistical analysis and its interpretation appropriate?

I cannot comment. A qualified statistician is required.

Are all the source data underlying the results available to ensure full reproducibility?

Yes

Is the study design appropriate and is the work technically sound?

Yes

Are the conclusions drawn adequately supported by the results?

Yes

Are sufficient details of methods and analysis provided to allow replication by others?

Partly

Reviewer Expertise:

Stroke , caregiver

I confirm that I have read this submission and believe that I have an appropriate level of expertise to confirm that it is of an acceptable scientific standard, however I have significant reservations, as outlined above.

# Response

**Published:** 2025-04-13

**Authors:** araya chaingkhong

**Affiliations:** Kuakarun Faculty of Nursing, Navaminradhiraj University, Bangkok, Thailand

We are grateful to Reviewer 2 for the insightful feedback that helped us clarify key aspects of our methodology and discussion.

 Methods

- Added explanation on how **confounding variables** were controlled: variables with p < 0.2 in univariate analysis were included in the adjusted logistic regression model.
- Clarified that **consecutive sampling** was used for eligible participants, and **random selection** was applied only for controls.
- Clarified that the study did not use randomization in an interventional sense, but used appropriate random selection methods for observational controls.
- Provided details on the **questionnaire validation** process: literature-based design, expert review, pilot testing, and Cronbach’s alpha reliability scores.

Discussion

- Rewrote parts of the discussion to offer **more critical reflection** and explicitly linked the findings (gender, sedentary behavior, marital status, workplace factors) to existing literature, especially regarding age-specific patterns.

Other Sections

- Confirmed that all **claims are now supported by appropriate, current references** throughout the manuscript.

# Reviewer report: Reviewer response for version 1

**DOI:** 10.5256/f1000research.170076.r358376

**Published:** 2025-02-19

**Authors:** Lamia M’barek

**Affiliations:** 1 Department of Neurology, China National Clinical Research Center for Neurological Diseases, Beijing Tiantan Hospital, Capital Medical University, Beijing, China

The study titled **"Lifestyle and Occupational Factors Associated with Recurrent Stroke among Working-Age Adults in Urban Areas of Thailand"** is a cohort study that includes ischemic stroke patients divided into two groups based on recurrent ischemic stroke. The primary aim is to identify significant factors associated with recurrent stroke in working-age adults in urban Thailand.

 The results indicate that female gender, high fasting blood sugar (FBS), drinking status, sedentary lifestyle, and lack of workplace support for health are significant factors associated with recurrent stroke. Additionally, the study highlights differences in the patterns of significant factors between younger and older workers.

 Overall, the results of this study are promising, and the discussion is clear. However, there are some comments for improvement to enhance the quality of the paper:

**Comments to Authors**

**Abstract:**

**Methods:** 

1. The study is described as a case-control study; however, it includes two groups: patients with recurrent ischemic stroke and those without recurrence. This design aligns more with a cohort study rather than a case-control study.
2. It may be beneficial to remove the control participants and focus the study on two groups based solely on recurrent stroke status.
3. **Matched Participants:** The authors state that patients are matched by age and gender. However, the distribution of gender is not matched (p < 0.05), as indicated in Table 1. Additionally, it would be helpful to present the age of all participants, regardless of group.
4. The number of patients should be clearly stated in the results section of the abstract.

**Results:**

1. There is missing data regarding the mean age and sex ratio of all participants.
2. Please specify that the odds ratio is adjusted (aOR) and present it in the following format: aOR: ..; 95% CI [..-..]; p: …

**Conclusion:**

1. Please cite the major results obtained from the study clearly.

**Main text**

**Introduction:**

1. Could the authors please clarify what they mean by “uncontrolled factors like alcohol consumption, diabetes, and hypertension”? Since these factors are modifiable, do the authors intend to suggest that there is inadequate control of these factors?
2. The term “working-age adults” should be defined, with references to previous studies in the same area.
3. The phrase “urban work-related stressors” is unclear and would benefit from further explanation.

**Methods:**

1. As previously mentioned in my comments on the abstract, the classification of the study as a case-control study should be reconsidered.
2. The age range referred to in this paragraph does not match Table 1. Are patients aged 20 to 25 missing, or is there a lack of patients in this age group? This needs clarification.
3. In the section on setting and sample, what is meant by "any severe complications"? Please specify that hemorrhagic and other cerebral damages are excluded.
4. The last sentence in this paragraph should be reformulated to emphasize the importance of stroke patients, avoiding the repetition of the phrase "300 patients."
5. In Figure 1, the label "stroke recurrence > 1" needs clarification. Does the study include only one recurrence? Additionally, Figure 1 would be more appropriately placed in the results section.
6. When stratifying participants into age groups, could the authors clarify which references they used for this division? This should be detailed in the Methods section.
7. What is the unit for FBS (Fasting Blood Sugar)? What do “normal,” “medium,” and “high” refer to? These values should be specified in the Methods section.
8. There seems to be a missing section regarding the follow-up of patients. How were patients followed? What was the duration and what kind of examinations were conducted?
9. Could the authors further clarify the Data Collection Procedure section? What is the distinction between this section and the Instruments section? Please ensure that there is no repetition between these two paragraphs.

**Result:**

1. There is missing data regarding the general population, including the mean age for both groups and the sex ratio. This information should be included for clarity.
2. The first paragraph lacks clarity; a further simplification is recommended to enhance understanding.
3. The terms “older workers” and “younger workers” were not defined in the Methods section. All relevant terms should be clearly defined in that section.
4. Regarding the adjusted odds ratio (aOR), the authors need to specify which factors were used for adjustment. This information should be included in the Methods section and referenced under the corresponding table.

**Conclusion **

1. It is essential to summarize the most crucial results of your work. These summaries should be presented concisely. In the conclusion section, ensure that you cite these results, as this will strengthen the credibility of your conclusions and provide a clear link between the findings and the overall message of your paper.

**Declarations**

1. The Declarations section appears to be repetitive. According to the journal's recommendations, please ensure that all relevant information is consolidated into a single section in the paper for clarity and compliance.

Is the work clearly and accurately presented and does it cite the current literature?

Yes

If applicable, is the statistical analysis and its interpretation appropriate?

Yes

Are all the source data underlying the results available to ensure full reproducibility?

Partly

Is the study design appropriate and is the work technically sound?

Partly

Are the conclusions drawn adequately supported by the results?

Yes

Are sufficient details of methods and analysis provided to allow replication by others?

Partly

Reviewer Expertise:

Clinical Research for Neurological Diseases

I confirm that I have read this submission and believe that I have an appropriate level of expertise to confirm that it is of an acceptable scientific standard, however I have significant reservations, as outlined above.

# Response

**Published:** 2025-04-13

**Authors:** araya chaingkhong

**Affiliations:** Kuakarun Faculty of Nursing, Navaminradhiraj University, Bangkok, Thailand

We sincerely thank Reviewer 1 for the detailed and constructive feedback, which has been instrumental in improving the clarity and rigor of our manuscript. Below are our point-by-point responses:

 Abstract

- We clarified that our study design is a case-control study, based on retrospective outcome status (recurrent vs. non-recurrent stroke), not a cohort design.
- Removed the incorrect reference to age/gender matching and revised the methodology accordingly.
- Moved the number of participants (100 cases, 200 controls) to the Results section of the abstract.
- Added mean age and sex ratio of both groups in the Results.
- Reworded odds ratios using adjusted format: aOR; 95% CI [lower–upper].
- Revised the conclusion in the abstract to directly cite significant findings.

Introduction

- Clarified that “uncontrolled factors” refer to **inadequately managed modifiable risk factors**.
- Defined “working-age adults” as aged 20–60 years and provided references.
- Expanded the explanation of “urban work-related stressors.”
- Reaffirmed that the case-control approach is appropriate and clearly stated this in the revised manuscript.

Methods

- Clarified that the age range discrepancy (20–24) is due to no eligible participants in that group.
- Specified “severe complications” as including hemorrhagic stroke, TIA, and other neurological impairments.
- Revised the final sentence to avoid redundancy in stating "300 patients."
- Clarified Figure 1 to indicate exclusion of >1 recurrence, and moved it to the Results section.
- Added references supporting age group classification (early-career: 25–40; mid-to-late-career: 41–60).
- Specified that FBS was measured in mg/dL using the oxidase enzymatic method and categorized using ADA guidelines.
- Explained follow-up procedures as retrospective record review, not active follow-up.
- Differentiated clearly between “Instruments” and “Data Collection” sections.

Results

- Added the mean age and sex ratio to improve demographic clarity.
- Revised the first paragraph for clarity and readability.
- Defined “younger” and “older” workers as “early-career” and “mid-to-late-career,” respectively, in alignment with occupational health literature.
- Specified all variables adjusted in the logistic regression model in both the Methods and Table 2 caption.

Discussion and Conclusion

- Enhanced the discussion with more critical analysis linking findings (e.g., gender, marital status, workplace support) to relevant literature.
- Rewrote the conclusion to summarize the most important results (with aORs) and directly support the study recommendations.

# Response

**Published:** 2025-04-13

**Authors:** araya chaingkhong

**Affiliations:** Kuakarun Faculty of Nursing, Navaminradhiraj University, Bangkok, Thailand

We sincerely thank Reviewer 1 for the detailed and constructive feedback, which has been instrumental in improving the clarity and rigor of our manuscript. Below are our point-by-point responses:

 Abstract

- We clarified that our study design is a case-control study, based on retrospective outcome status (recurrent vs. non-recurrent stroke), not a cohort design.
- Removed the incorrect reference to age/gender matching and revised the methodology accordingly.
- Moved the number of participants (100 cases, 200 controls) to the Results section of the abstract.
- Added mean age and sex ratio of both groups in the Results.
- Reworded odds ratios using adjusted format: aOR; 95% CI [lower–upper].
- Revised the conclusion in the abstract to directly cite significant findings.

Introduction

- Clarified that “uncontrolled factors” refer to **inadequately managed modifiable risk factors**.
- Defined “working-age adults” as aged 20–60 years and provided references.
- Expanded the explanation of “urban work-related stressors.”
- Reaffirmed that the case-control approach is appropriate and clearly stated this in the revised manuscript.

Methods

- Clarified that the age range discrepancy (20–24) is due to no eligible participants in that group.
- Specified “severe complications” as including hemorrhagic stroke, TIA, and other neurological impairments.
- Revised the final sentence to avoid redundancy in stating "300 patients."
- Clarified Figure 1 to indicate exclusion of >1 recurrence, and moved it to the Results section.
- Added references supporting age group classification (early-career: 25–40; mid-to-late-career: 41–60).
- Specified that FBS was measured in mg/dL using the oxidase enzymatic method and categorized using ADA guidelines.
- Explained follow-up procedures as retrospective record review, not active follow-up.
- Differentiated clearly between “Instruments” and “Data Collection” sections.

Results

- Added the mean age and sex ratio to improve demographic clarity.
- Revised the first paragraph for clarity and readability.
- Defined “younger” and “older” workers as “early-career” and “mid-to-late-career,” respectively, in alignment with occupational health literature.
- Specified all variables adjusted in the logistic regression model in both the Methods and Table 2 caption.

Discussion and Conclusion

- Enhanced the discussion with more critical analysis linking findings (e.g., gender, marital status, workplace support) to relevant literature.
- Rewrote the conclusion to summarize the most important results (with aORs) and directly support the study recommendations.

Other Sections

- Consolidated the “Declarations” section for clarity and to meet journal requirements.
